# JNK1 Derived from Orange-Spotted Grouper, *Epinephelus coioides*, Involving in the Evasion and Infection of Singapore Grouper Iridovirus (SGIV)

**DOI:** 10.3389/fmicb.2016.00121

**Published:** 2016-02-10

**Authors:** Minglan Guo, Jingguang Wei, Xiaohong Huang, Yongcan Zhou, Yang Yan, Qiwei Qin

**Affiliations:** ^1^Key Laboratory of Tropical Marine Bio-resources and Ecology, South China Sea Institute of Oceanology, Chinese Academy of SciencesGuangzhou, China; ^2^Guangdong Provincial Key Laboratory of Applied Marine Biology, South China Sea Institute of Oceanology, Chinese Academy of SciencesGuangzhou, China; ^3^Key Laboratory of Tropical Biological Resources of Ministry of Education, Hainan UniversityHaikou, China; ^4^Laboratory for Marine Biology and Biotechnology, Qingdao National Laboratory for Marine Science and TechnologyQingdao, China

**Keywords:** apoptosis, c-Jun N-terminal kinases 1, *Epinephelus coioides*, replication, Singapore grouper iridovirus (SGIV), virus infection

## Abstract

c-Jun N-terminal kinase (JNK) regulates cellular responses to various extracellular stimuli, environmental stresses, pathogen infections, and apoptotic agents. Here, a JNK1, Ec-JNK1, was identified from orange-spotted grouper, *Epinephelus coioides*. Ec-JNK1 has been found involving in the immune response to pathogen challenges *in vivo*, and the infection of Singapore grouper iridovirus (SGIV) and SGIV-induced apoptosis *in vitro*. SGIV infection activated Ec-JNK1, of which phosphorylation of motif TPY is crucial for its activity. Over-expressing Ec-JNK1 phosphorylated transcription factors c-Jun and promoted the infection and replication of SGIV, while partial inhibition of the phosphorylation of Ec-JNK1 showed the opposite effects by over-expressing the dominant-negative EcJNK1-Δ183-185 mutant. Interestingly, SGIV enhanced the viral infectivity by activating Ec-JNK1 which in turn drastically inhibited the antiviral responses of type 1 IFN, indicating that Ec-JNK1 could be involved in blocking IFN signaling during SGIV infection. In addition, Ec-JNK1 enhanced the activation of AP-1, p53, and NF-κB, and resulted in increasing the levels of SGIV-induced cell death. The caspase 3-dependent activation correlated with the phosphorylation of Ec-JNK1 and contributed to SGIV-induced apoptosis. Taken together, SGIV modulated the phosphorylation of Ec-JNK1 to inactivate the antiviral signaling, enhance the SGIV-induced apoptosis and activate transcription factors for efficient infection and replication. The “positive cooperativity” molecular mechanism mediated by Ec-JNK1 contributes to the successful evasion and infection of iridovirus pathogenesis.

## Introduction

The c-Jun NH2-terminal kinases (JNKs) are one subgroup of the mitogen-activated protein kinases (MAPKs) family (Davis, 2000; Chang and Karin, 2001; Weston and Davis, 2007). JNKs can be activated by various extracellular stimuli, including inflammatory cytokines, environmental stress, UV irradiation, and pathogen infections (Davis, 2000; Raman et al., 2007; Li et al., 2012). JNK is required for many aspects of normal cellular physiology. In mammalian, JNK has been implicated in the immune response (Rincón et al., 2001; Zhu et al., 2012), oncogenic transformation (Accardi et al., 2013), cell survival (Davis, 2000; Hrincius et al., 2010), tumor development (Kennedy and Davis, 2003), and apoptosis (Verheij et al., 1996; Suzuki and Matsuoka, 2013). In fishes, JNK molecules play crucial roles in embryonic development and organogenesis (Seo et al., 2010; Xiao et al., 2013), and regulation of innate immune (Taylor et al., 2013). However, the roles and mechanisms of JNK molecules in pathogen infection are still uncertain.

Viruses have been known to utilize various cellular signaling pathways to achieve infection and replication (Nguyen et al., 2007). Recently, the JNK signaling pathway has been shown to be involved in various viral infections, such as Rotavirus (Holloway and Coulson, 2006), Coxsackievirus B3 (Si et al., 2005), Human immunodeficiency virus type 1(HIV-1) (Muthumani et al., 2004), Influenza virus (Hrincius et al., 2010), varicella-zoster virus (Zapata et al., 2007), Herpes simplex virus (HSV) (McLean and Bachenheimer, 1999), Bombyx mori Nucleopolyhedrovirus (Katsuma et al., 2007), Murine gammaherpesvirus 68 (Stahl et al., 2012), and Singapore grouper iridovirus (SGIV) (Huang et al., 2011a). SGIV is a major cause of mortality in fishes, such as grouper and seabass (Qin et al., 2003; Huang et al., 2004). The mechanisms of SGIV infection remain largely unknown, and it appears that multiple factors and cellular signaling pathways are involved in the infection of SGIV (Huang et al., 2011c). Our previous study showed that JNK pathway is important for SGIV replication and modulates the inflammatory responses during virus infection (Huang et al., 2011a). Phosphorylation of JNK1/2 and p38 MAPK and activation of its downstream effectors occurred during active replication of SGIV in grouper cell. However, the cytopathic mechanisms of JNK molecules were unclear, and its role in the infection and replication of SGIV has not yet been elucidated.

JNK molecules show multiple functions in virus infection. JNK1 and JNK2 signaling pathways play distinct roles in cell-mediated antiviral immunity (Arbour et al., 2002). Antiviral effect of CDF40 requires activation of JNK1/2 (Rau et al., 2013). Influenza A virus (IAV) protein A/NS1 activates the JNK that in turn mediates antiviral responses to IAV infection (Hrincius et al., 2010). However, JNK molecules are also activated by virus infection, and play important roles in viral infection and replication (McLean and Bachenheimer, 1999; Si et al., 2005; Shi et al., 2012). The activity of JNK has been linked to virus infection and replication mainly through the effect of downstream transcription factors, such as c-Jun (Lee et al., 2011; Stahl et al., 2012), activator protein 1 (AP-1) (Tardif and Tremblay, 2005; Presser et al., 2013) and NF-κB (Thome et al., 1999; Shi et al., 2012) on transcriptional regulation of viral genes.

Apoptosis is essential for the maintenance of homeostasis in the immune system. And apoptotic cell death occurs in a wide range of viral infections (Clarke and Tyler, 2009; Kinpara et al., 2013). To ensure their own survival and propagation, viruses modulate the crucial aspects of host homeostasis, influence the cell cycle and regulate the apoptotic machinery (Tortorella et al., 2000; Gougeon and Piacentini, 2009). Virus-induced apoptosis has been found to be related with activation of c-Jun N-terminal kinase (Hrincius et al., 2010; Shi et al., 2012), NF-κB, and p53 pathways (Myskiw et al., 2009; Kinpara et al., 2013). As described previously, SGIV can induce the apoptosis in individual cells (Huang et al., 2011b). However, little information is known about the mechanism of SGIV-induced apoptosis, and the roles of JNK molecules are not well established in the apoptosis.

Orange-spotted grouper, *Epinephelus coioides*, has served as a good model for studying protection against viral infection of *iridovirus* (Mahardika et al., 2004; Yeh et al., 2008; Huang et al., 2011c; Ou-yang et al., 2012a) and *betanodavirus* (Kai and Chi, 2008; Wu et al., 2012; Yeh et al., 2014) in teleost fish, and contributed to understand interaction of fish cells with marine *iridovirus* (Qin et al., 2006; Guo et al., 2012) and *betanodavirus* (Chi et al., 1999; Parameswaran et al., 2007). In this study, a JNK1 molecule, Ec-JNK1, was derived from grouper *E. coioides*. The characterization and roles of Ec-JNK1 on iridovirus infection of SGIV were investigated. Data from dominant-active and dominant-negative Ec-JNK1 focusing on the Thr-Pro-Tyr (TPY) motif demonstrated that SGIV activated Ec-JNK1 to facilitate the efficient infection. Ec-JNK1 promoted SGIV infection and replication, and the SGIV-induced apoptosis. However, dominant-negative EcJNK1-Δ183-185 mutant, which partially inhibited the activation of Ec-JNK1, showed the opposite effects. SGIV infection enhanced the activation of Ec-JNK1, which suppressed the expression of type 1 IFN, and resulted in increasing viral infectivity. The caspase 3-dependent activation positively related to the phosphorylation of Ec-JNK1. Ec-JNK1 activated AP-1, p53, and NF-κB, and increased the levels of SGIV-induced apoptosis. All results demonstrated that a “positive cooperativity” molecular mechanism mediated by Ec-JNK1 contributed to the successful evasion and infection of iridovirus pathogenesis.

## Materials and methods

### Fish, virus, and cells

Juvenile orange-spotted grouper, *E. coioides* (length 6–10 cm, weight 15–30 g) were purchased from a marine-culture farm at Honghai bay, Shanwei City, Guangdong Province, China. Fishes were cultured 2 weeks in laboratory and treated as described previously (Guo et al., 2012) for collection of tissues. In brief, a series of immune-related tissues, including liver, spleen, kidney, brain, intestine, heart, skin, muscle, gill, stomach, and head kidney, were sampled for mRNA extraction. After challenging with SGIV (5 × 10^5^ TCID_50_/ml, tissue culture infective dose), Lipopolysaccharide (LPS, 15 mg/kg i.v., Sigma-Aldrich, #L2880), and Polyriboinosinic Polyribocytidylic Acid (poly I:C, 10 mg/kg i.v., Sigma-Aldrich, #P9582), tissues of liver and spleen were dissected from five individuals at indicated times of post-injection (0, 1, 2, 6, 12, 24, 48, 72, and 96 h). This study involving animals was carried out in accordance with the recommendations of “guidelines of the Animal Care and Ethical Committee, South China Sea Institute of Oceanology, Chinese Academy of Sciences.” Each group of the samples contained five individuals to eliminate individual differences. Samples from five individuals were put together and immediately frozen by liquid nitrogen and stored at −80°C until the total RNA extraction. The protocol was approved by the South China Sea Institute of Oceanology, Chinese Academy of Sciences.

The virus SGIV (strain A3/12/98 PPD) used in the study was propagated as described in the literature (Qin et al., 2001). Two fish cell lines, grouper spleen (GS) (Qin et al., 2006) and fathead minnow (FHM) epithelial cells (Gravell and Malsberger, 1965), were grown in Leibovitz's L15 medium and M199 medium containing 10% fetal bovine serum (Invitrogen, USA) at 25°C, respectively.

### RNA extraction and cDNA synthesis

Tissues of each group were incubated with liquid nitrogen, transferred to a mortar (RNase free) and ground to a fine powder. TRIzol reagent were then used to save samples. Total RNA of grouper tissues was extracted using TRIzol reagent (Invitrogen) according to the manufacturer's protocol. Agarose gel electrophoresis was performed to examine the quality of total RNA. Then, total RNA was transcribed to cDNA by ReverTra Ace Kit (TOYOBO, Japan). First-strand cDNAs for 3′RACE and 5′RACE were synthesized from total liver RNA with SMART™ RACE cDNA amplification kit (Clontech, USA).

### Cloning and sequencing JNK1 from *E. coioides* (Ec-JNK1)

To identify grouper JNK1 cDNA sequence, primers Ec-JNK1F1 and Ec-JNK1R1 (Table 1) were designed by comparing the known fish JNK1 sequences, such as *Danio rerio* (NM_131721.1), *Monopterus albus* (EF661977.1), and *Carassius auratus* (EU374209.1). Primers Ec-JNK1F1 and Ec-JNK1R1 were used to amplify the partial sequence of Ec-JNK1 from liver cDNA. According to the partial sequence, primers 3′EcJNK1F1 and 3′EcJNK1F2, 5′EcJNK1R1 and 5′EcJNK1R2 (Table 1) were designed to obtained the full-length cDNA of Ec-JNK1 from first-strand cDNAs of 3′RACE and 5′RACE according to the manufacturer's protocol of SMART™ RACE cDNA amplification kit. BioEdit and Expasy search program (http://au.exasy.org/tools/) was used to analyze the nucleotide and predicted amino acid sequences of Ec-JNK1. SMART program (http://smart.embl-heidelberg.de/) was used to predict the domain structure. The BLASTP search program at the NCBI (http://www.ncbi.nlm.nih.gov/blast) was performed to analyze the similarity of Ec-JNK1 with other JNK1s. Clustalx 1.83(http://www.ebi.ac.uk/clustalW/) and MEGA 4.0 software (http://megasoftware.net/) were used for the multiple-sequence alignment of the reported JNK1 amino acid sequences and construction of phylogenetic tree, respectively.

**Table 1:** **Sequences of primers used in this study**.

**Primers**	**Sequence (5′-3′)**
Ec-JNK1F1	ATGAAC(A/C)GGAA(T/C)AAGCGCGAG
Ec-JNK1R1	AGAGATCCGCTT(G/A)GACGCATC(T/G)ATTACC
3′EcJNK1F1	GAGGCTCTCCAGCACCCTTATATC
3′EcJNK1F2	GAAGTGTTGGACTGGGAAGAAAGGAC
5′EcJNK1R1	TGGCAGAGGTTGGCATCCATCAGC
5′EcJNK1R2	AGCACCAGTTCCCTGTAAGCCCGT
pET32a-EcJNK1F	CGGATATCATGAACAGGAACAAGCG
pET32a-EcJNK1R	CCCAAGCTTTCACTGTTGCACCTGT
pcDNA-EcJNK1F	GGGGTACCATGAACCGGAACAAGCGGCGA
pcDNA-EcJNK1R	GCTCTAGATCACTGTTGCACCTGT
EcJNK1mutantF	ATGGCGCCCTTCGTGGTCACCCGCTACTACC
EcJNK1mutantR	CACGAAGGGCGCCATGAGGAGGCCGGTGGCA
RT-EcJNK1F	CGGGGTAGTGTGTTGTTTCCA
RT-EcJNK1R	GGCTCGCTTTCAGTTTGTTGT
RT-pcDNAF	GGCACCAAAATCAACGGGACTTTC
RT-pcDNAR	AGCAGTGGGTTCTCTAGTTAGCC
ActinF	TACGAGCTGCCTGACGGACA
ActinR	GGCTGTGATCTCCTTTTGCA

*The underlined text means enzyme digestion sites (pET32a-EcJNK1F, pET32a-EcJNK1R, pcDNA-EcJNK1F, pcDNA-EcJNK1R) and mutant sites (EcJNK1mutantF, EcJNK1mutantR) added in the designing primers*.

### Expression profiles of Ec-JNK1 *in vivo*

Primers RT-EcJNK1F and RT-EcJNK1R (Table 1) were used for expression profiles of Ec-JNK1 in tissue distribution and pathogenic challenge by Real-time quantitative PCR (RT-qPCR). Primers ActinF and ActinR (Table 1) were used to amplify the internal control β-actin. The cDNAs of each group were used as the template. RT-qPCR was performed on Roche LightCycler® 480 Real-time PCR system (Roche, Switzerland), using “2 × SYBR Green Real-time PCR Mix” (TOYOBO, Japan). Cycling conditions were used as follows: 95°C for 3 min, followed by 40 cycles of 5 s at 94°C,10 s at 55°C, and 15 s at 72°C. The expression assay was performed in triplicate. All data were analyzed as previously described (Guo et al., 2012), normalized relative to β-actin expression, and given in terms of relative mRNA expression level as means ± standard deviation (S.D.). For challenge assays, the data among different times were statistically analyzed using the multiple comparisons of LSD and Duncan methods containing in One-Way ANOVA of software SPSS 16.0.

### Expression of recombinant protein and preparation of antiserum

Primers pET32a-EcJNK1F and pET32a-EcJNK1R (Table 1) were used to amplify the open reading frame (ORF) region of Ec-JNK1 from grouper liver cDNA. The target sequence was cloned in vector pET32-a (+) (Novagen, Germany). Plasmid pET32a-EcJNK1 was transformed into *Escherichia coli* BL21 (DE3). Recombinant protein was expressed under condition of isopropyl-1-thio-β-D-galactopyranoside (IPTG, 1.0 mM; Merke, Germany) at 16°C, 200 rpm for 10 h. The protein Ec-JNK1 was purified from inclusion bodies of recombinant *E. coli* and solubilized with 6 mol l^−1^ guanidinium hydrochloride, 0.1 mol l^−1^ Tris-HCl (pH 8.5), 0.1 mol l^−1^ dithiothreitol, 1 mmol l^−1^ EDTA (Müller and Rinas, 1999). The protein concentration of Ec-JNK1 was quantified with phosphate buffer saline (PBS). The polyclonal antibody of Ec-JNK1 was produced by immunizing BALB/c mice according to the conventional method (Sambrook et al., 1989) and collected for the following experiment. The control, pre-immune mouse serum, was obtained after skin injection with PBS. Twelve percent sodium dodecyl sulfate-polyacrylamide gel electrophoresis (SDS–PAGE) was performed to examine the recombinant protein of Ec-JNK1. To detecting the specificity of the obtained antibody of Ec-JNK1, the purified protein and GS cell lysate were separated and used to detect obtained Ec-JNK1 antibody by western blot as described previously (Guo et al., 2012). The mouse anti-EcJNK1 serum and pre-immune mouse serum diluted to 1:2000 were used as primary antibody, and HRP-conjugated goat anti-mouse antibody (Pierce, USA) was served as secondary antibody at a dilution of 1:2000. In addition, three screening cell lines, FHM/pcDNA-EcJNK1, FHM/pcDNA-EcJNK1-Δ183-185, and FHM/pcDNA3.1 (5 × 10^5^), were separated by 12% SDS-PAGE and detected with the antibodies above by western blot to confirm the specificity of anti-EcJNK1 serum and define the screening cell lines. The β-actin was used as the internal controls and examined with Rabbit anti-β-actin antibody (Cell Signaling, 13E5, #4970) at a dilution of 1:1000. HRP-labeled goat anti-Rabbit antibody (Pierce, USA) at a dilution of 1:1000 was used as the secondary antibody.

### Immunofluorescence staining

Immunofluorescence staining was performed to examine the sub-cellular localization of Ec-JNK1. GS cells (5 × 10^5^) were either mock- or infected with SGIV (0.5 MOI). At 0, 2, and 24 h post-infection (p.i.), GS cells were fixed in 4% buffered paraformaldehyde at 4°C for 2 h, permeabilized for 15 min and blocked with 2% bovine serum albumin (BSA) for 30 min. Ec-JNK1 in GS cells was fluorescently labeled using polyclonal antibody of Ec-JNK1 (1:100) for 2 h and FITC-conjugated goat anti-mouse IgG antibody (Pierce, USA) for 1 h. Nuclei or viral DNA were stained with 4′, 6-diamidino-2-phenylindole (DAPI, 1 μg/mL; Sigma, USA). Staining cells were then undergone fluorescence microscopic observation. To confirm the specificity of anti-EcJNK1 serum and sub-cellular localization of Ec-JNK1, mock-, or infected (24 h p.i.) GS cells were immunofluorescently stained with the control pre-immune mouse serum as methods above.

### Over-expression of Ec-JNK1 and dominant-negative EcJNK1-Δ183-185 mutant

The plasmid pcDNA-EcJNK1 was constructed in vector pcDNA3.1 (+) (Invitrogen, USA) using primers pcDNA-EcJNK1F and pcDNA-EcJNK1R (Table 1). Site-directed mutagenesis was carried out to generate the TPY dominant-negative mutant of EcJNK1-Δ183-185 using the overlap-PCR method (Heckman and Pease, 2007). Threonine 183 and tyrosine 185 of Ec-JNK1 were replaced with alanine and phenylalanine using primers pcDNA-EcJNK1F and EcJNK1mutantR, EcJNK1mutantF and pcDNA-EcJNK1R (Table 1), respectively. The mutant fragment of EcJNK1-Δ183-185 was cloned in pcDNA3.1 (+) vector. Then, plasmids of pcDNA-EcJNK1, pcDNA-EcJNK1-Δ183-185 and pcDNA 3.1 (+) (the control) were confirmed by sequencing and transfected into FHM cells as methods described previously (Guo et al., 2012). Screening cell lines stably expressing the three plasmids were obtained by selecting with 400 ng/mL G418 (Sigma-Aldrich, USA) for 4 weeks.

### Activities of Ec-JNK1 and dominant-negative EcJNK1-Δ183-185 mutant *in vitro*

To study the role of Ec-JNK1, cells of FHM/pcDNA-EcJNK1, FHM/pcDNA-EcJNK1-Δ183-185, and FHM/pcDNA3.1 (5 × 10^5^) were either mock- or infected with SGIV (0.5 MOI). Transcriptional analysis was performed to define the expression levels of both wild type and mutant Ec-JNK1. In brief, mock- cell samples above were harvested for RNA extraction and cDNA synthesis, and then the expression profiles of vector pcDNA3.1 and Ec-JNK1 which were assessed by qRT-PCR with primers RT-pcDNAF and RT-pcDNAR, RT-EcJNK1F, and RT-EcJNK1R (Table 1). β–actin was amplified as internal control. The quantitative mRNA expressions were assessed by electrophoresis on 1.2% agarose gel. In addition, western blot above has been performed to define the screening cell lines. In parallel, mock- and infected cells (2, 6, 12, 24, and 48 h p.i.) were collected and re-suspended in PBS containing 1 × SDS loading buffer. Phosphorylation of JNK and transcription factors c-Jun was examined by western blot. Anti-phospho-JNK/SAPK (Cell Signaling, #4668), anti-SAPK/JNK (Cell Signaling, #9252), anti-phospho-c-Jun (Cell Signaling, #9261) and anti-c-Jun (Cell Signaling, #9165) antibodies at a dilution of 1:1000 were used as the primary antibody, respectively, and detected with HRP-labeled goat anti-Rabbit antibody as method above. Immunoreactive bands were visualized with SuperSignal West Pico chemiluminescent substrate (Pierce, USA), followed by autoradiagraphy. Simultaneously, the productions of SGIV immediate-early gene ORF086R involved in cell growth control and virus replication (Xia et al., 2009), and SGIV early gene ORF136 associated with mitochondria (Huang et al., 2008), were detected by western blot to reveal the infection process of SGIV. β-actin was detected as internal controls.

At 24 and 48 h p.i., mock- or infected cells were observed by microscopy (Leica). All cells were harvested to examine the replication of SGIV. Equal amounts of cell lysate extracts were subjected to the SDS-PAGE and western blot analyses. The viral structure proteins of late stage gene ORF39L and ORF072 were detected using mouse anti-SGIV ORF39L serum (Zhang et al., 2015) and anti-SGIV ORF 072 serum (Ou-yang et al., 2012b) at a dilution of 1:2000, respectively. The internal controls were β-actin that examined as method above. In parallel, the cell viability was determined using the trypan blue exclusion test. Mock- or infected cells were collected and incubated in 0.2% trypan solution (Sigma) for 5 min. The dead cells of each sample were counted by three independent hemocytometer counts. The cell viability was presented as percentage of viable cells over total number of cells. At 24 and 48 h p.i., supernatants and infected cell lysates were collected to determine the viral titer 50% tissue culture infectious dose (TCID_50_) (Reed and Muench, 1938). Each experiment was repeated three times with each comprising triplicates.

### Effect of Ec-JNK1 and dominant-negative Ec-JNK1-Δ183-185 mutant on SGIV-induced apoptosis

To determine the function of Ec-JNK1 on SGIV-induced apoptosis, three FHM cells above (5 × 10^5^) were plated onto glass cover slips in six well plates and inoculated with SGIV or PBS. At 24 h p.i., the cells were washed with PBS and incubated with 4% paraformaldehyde for 1 h. After fixation, cells were stained with 1 μg/ml Hoechst 33258 (Sigma) in PBS for 5 min at room temperature. After washing with PBS, slips of nucleus staining were observed under fluorescent microscope (Leica).

The percentages of apoptotic cells were quantified by flow cytometry analyses as described previously (Huang et al., 2011b). Briefly, mock- or infected cells were harvested and fixed in 70% ice-cold ethanol overnight at −20°C. After washing with PBS, the cells were centrifuged at 500 × g and incubated for 30 min in PBS containing propidium iodide (PI, 50 μg/mL; Sigma) and DNase-free RNase A (100 μg/mL; Sigma). Cells (4 × 10^4^) stained with PI fluorescence were measured with a FAC-Scan flow cytometer (Becton-Dickinson). The obtained data was analyzed using the Cellquest software (Becton-Dickinson).

The changes of mitochondrial membrane potential (ΔΨm), the central point of apoptosis (Desagher and Martinou, 2000; Kroemer et al., 2007), were evaluated during SGIV infection. Cells of FHM/pcDNA-EcJNK1, FHM/pcDNA-EcJNK1-Δ183-185, and FHM/pcDNA3.1 (5 × 10^5^), were stained with JC-1 (Molecular Probes, #T3168) according to the manufacturer's instructions. At 24 h p.i., mock- and infected cells were washed with PBS and incubated with JC-1 (15 μg/mL) solution for 20 min. Then, the incubation buffer was replaced with fresh medium. Cells were immediately observed by the fluorescent microscope (Leica). JC-1, a lipophilic and cationic dye, can selectively accumulate into mitochondria and reversibly change color from green to red as the membrane potential increases. Mitochondrial depolarization is indicated by a decrease in the red/green fluorescence ratio. Flow cytometry analyses were performed to determine the percentages of cells with decreased ΔΨm. Mock- and infected cells (5 × 10^5^) were stained with JC-1 (15 μg/mL) solution for 15 min and centrifuged at 500 × g for 10 min. The cells were re-suspended in 0.5 ml cell culture medium. Staining cells (4 × 10^4^) were examined with the flow cytometer according to the manufacturer's protocol.

Caspase-3 plays typical role in apoptosis. The activities of caspase-3 were detected using fluorometric protease assay kits (BioVision) according to the manufacturer's protocol. Mock- and infected cells (5 × 10^5^) were harvested at 3, 6, 12, 18, 24, and 48 h p.i., and homogenized in lysis buffer. The cell lysates were stored at −80°C. The lysates were thawed on ice for 10 min, centrifuged at 10,000 × g at 4°C for 2 min. Then the supernatant was transferred to a fresh tube and put on ice. An equal concentration protein was used for each sample. All samples were added with the reaction buffer and incubated at 37°C for 2 h. A multifunctional microplate reader (Victor X5; PerkinElmer), which was equipped with a 400 nm excitation filter and 505 nm emission filter, was used to detected the caspase-3 activity. The data were expressed as fold-increase by comparing these results with the level of the mock-infected cells.

### Reporter gene assays

Plasmids of AP-1-Luc, p53-Luc, NF-κB-Luc, ISRE-Luc (Clontech, USA), and INF-Luc (Cui et al., 2011) were used to measure the activations of reporter genes AP-1, p53, NF-κB, IFN, and ISRE during the SGIV infection. In brief, FHM cells (5 × 10^5^) were co-transfected with 200 ng of plasmids above, 100 ng of pSV-β-galactosidase vector (Promega, USA) and 400 ng of pcDNA-EcJNK1, or pcDNA-EcJNK1-Δ183-185, or pcDNA3.1 using LipofectamineTM 2000 Reagent. At 16 h post-transfection, FHM cells were either mock- or infected with SGIV (at 0.5 MOI) for 48 h. Then, the cells were collected for the following test. Activities of luciferase and pSV-β-galactosidase of total cell lysates were detected with Luciferase assay system (Promega, USA). Luciferase activities were normalized to β-galactosidase enzymatic activity.

### Statistical analyses

All assays were carried out for three times. Results were represented as means ± S.D. Software SPSS 16.0 was used for statistical analysis of all data. In brief, data were statistically analyzed using Student's *t*-test. One-Way ANOVA was performed to analyze data over two groups. Multiple comparisons of LSD and Duncan were chosen as post hoc tests. The results of LSD and Duncan methods were consistent. The values of *P* < 0.05 and *P* < 0.01 were presented as statistically significant and highly significant, respectively. Statistic differences were indicated as ^*^p < 0.05; ^***^p < 0.01.

## Results

### Sequence analysis of Ec-JNK1

The full-length cDNA of Ec-JNK1 (GenBank accession no.KJ858685) was 1793 bp in length, which contained a 322 bp 5′ terminal untranslated region (UTR), 316 bp 3′UTR, and a 1155 bp ORF encoding a putative protein of 384 amino acids (GenBank accession no. AIK19653) residues with predicted molecular mass of 44.2 kDa. Position 26–321 of the predicted amino acid sequence was the domain of Serine/Threonine protein kinase (S_TKc), which contained a conserved dual phosphorylation motif TPY at the position of 183–185 (Figures S1, S2). Similar to other reported JNK1 from vertebrates, catalytic domain of the Serine/Thronine kinase, c-Jun N-terminal kinase (STKc_JNK) was at the position 25–360, among which contained a Serine/Threonine protein kinases active site signature (Figure S2). The JNK1 subfamily of Osteichthyes was conserved according to the alignment of the JNK1 amino acid sequences in various species. Ec-JNK1 shared 99% identity to *Pundamilia nyererei*. The phylogenetic tree of vertebrate JNK1 consisted of two major branches, and Ec-JNK1 was clustered into the Osteichthyes branch. Chondrichthyes were more closely related with amphibian and mammals (Figure S3).

### Expression profiles of Ec-JNK1 *in vivo*

**Figure 1:**
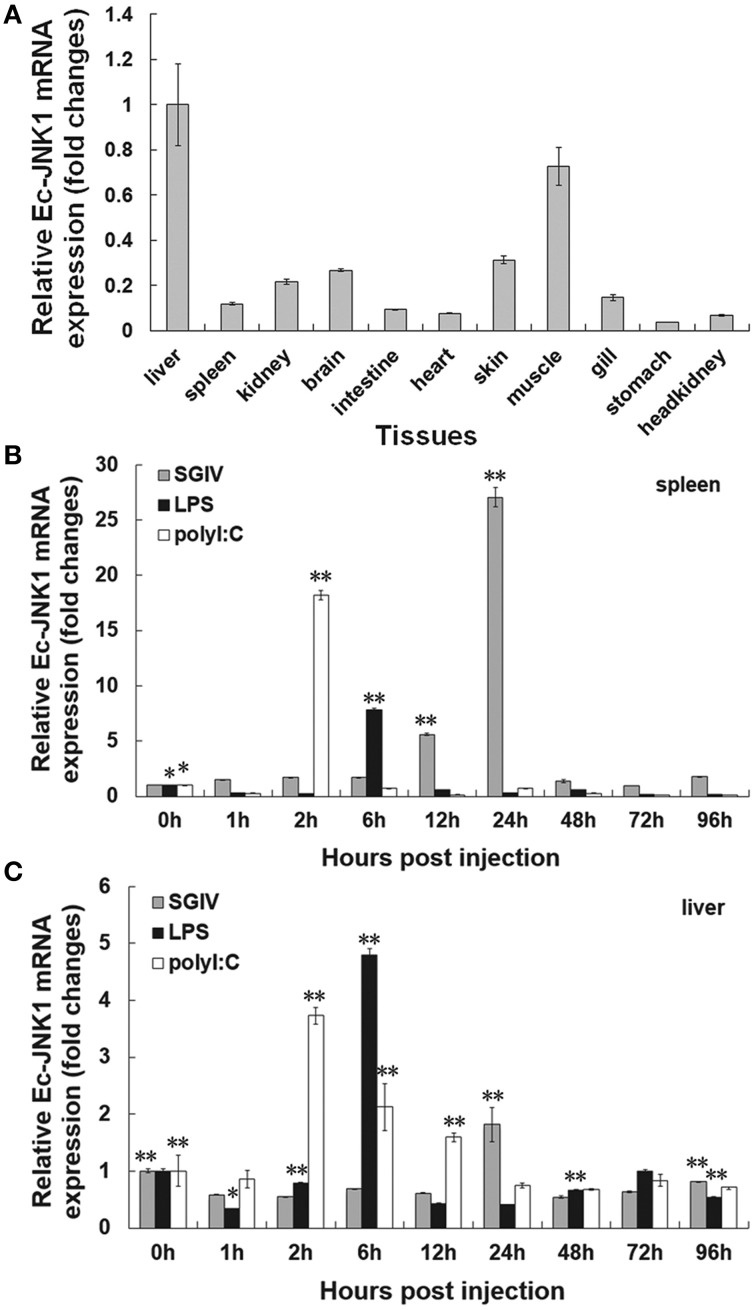
**Expression profiles of Ec-JNK1 *in vivo* quantified by RT-qPCR**. **(A)** Tissue distribution of Ec-JNK1 in juvenile orange-spotted grouper. Immune responses of Ec-JNK1 in spleen **(B)** and liver **(C)** after groupers challenged with LPS, SGIV, and poly I:C, respectively. The experiments were done in triplicate. All data were normalized relative to β–actin and represented by means ± standard deviation (S.D.) (*n* = 3). Statistical analyses among different times of pathogenic challenge were performed using multiple comparisons of LSD and Dancan in One-Way ANOVA of software SPSS 16.0. ^*^p < 0.05; ^**^p < 0.01.

A constitutive expression of Ec-JNK1 was distributed in all tissues examined in orange-spotted grouper (Figure 1A). Ec-JNK1 was expressed a high amount in liver, muscle, skin, brain, kidney, and gill, while a few in spleen, intestine, heart, head kidney and stomach. To explore the immune responses of Ec-JNK1 in pathogenic challenges, the relative expression of Ec-JNK1 were analyzed after the fish was injected with LPS, SGIV and poly I:C. Ec-JNK1 transcripts were significantly (*P* < 0.01) up-regulated and peaked at different post-injection times (LPS at 6 h, SGIV at 24 h, and poly I:C at 2 h) in both spleen and liver (Figures 1B,C).

### Expression of recombinant protein and preparation of antibody for Ec-JNK1

**Figure 2:**
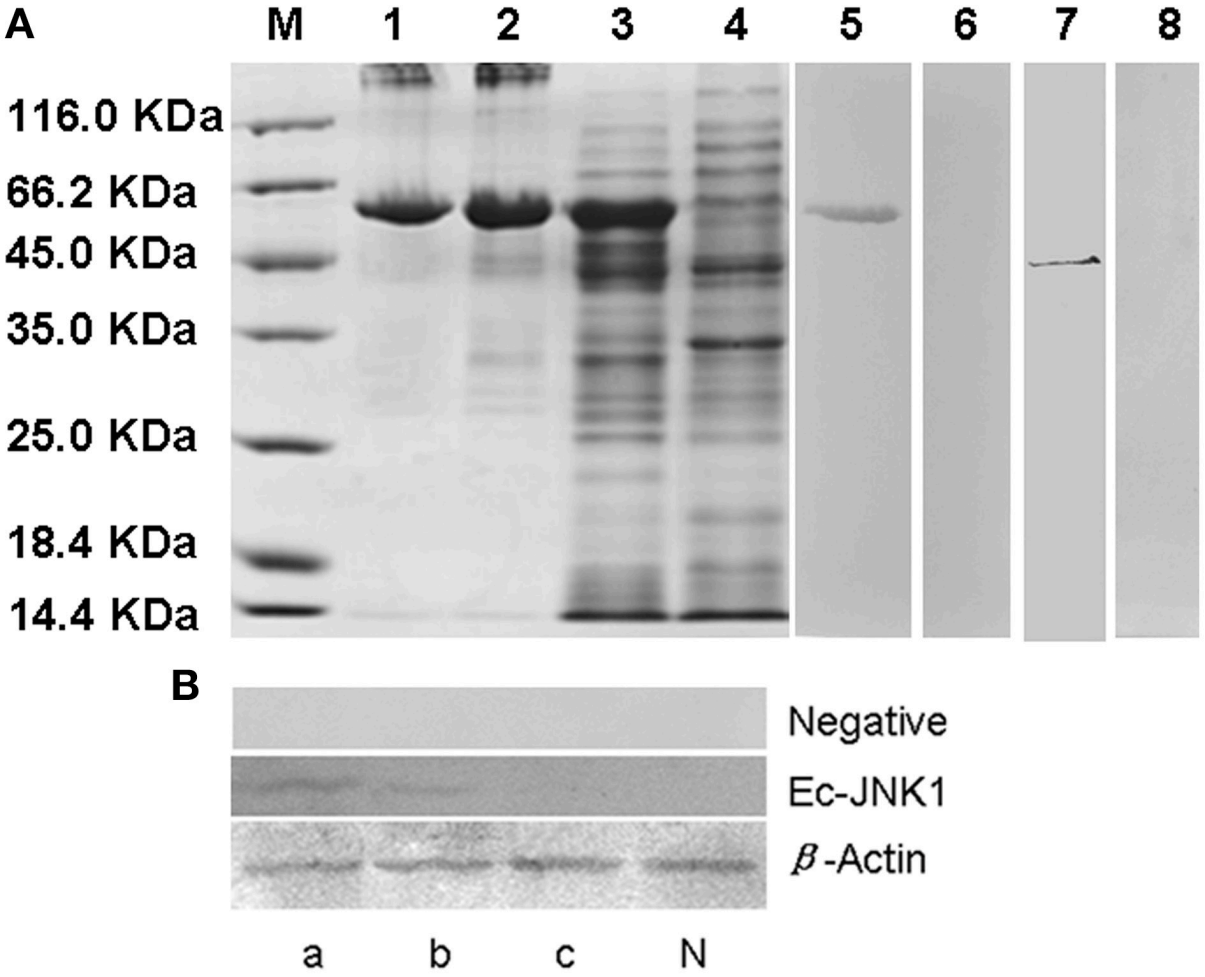
**Detection for recombinant protein and antibody of Ec-JNK1**. **(A)** Protein expression and antibody preparation for Ec-JNK1. M, Protein marker; 1, purified protein of Ec-JNK1; 2, sediment of induced product for pET-32a-EcJNK1; 3, pET-32a-EcJNK1 in BL21 (DE3), IPTG induced for 12 h; 4, pET-32a (+) expressed product; 5, 6, purified protein of Ec-JNK1 was detected with mouse anti-EcJNK1 serum and pre-immune mouse serum by western blot, respectively; 7, 8, GS cell lysate was used to detect the specificity of antibody with mouse anti-EcJNK1 serum and pre-immune mouse serum by western blot, respectively. **(B)** Mouse anti-EcJNK1 serum (line Ec-JNK1) and pre-immune mouse serum (line negative) were used to detect FHM cells stably expressing pcDNA-EcJNK1, pcDNA-EcJNK1-Δ183-185 mutant, and pcDNA3.1 (control). a, FHM/pcDNA-EcJNK1; b, FHM/pcDNA-EcJNK1-Δ183-185; c, FHM/pcDNA3.1; N, FHM.

Positive clone of *E. coli* BL21 containing plasmid pET32a-EcJNK1 successfully expressed recombinant protein of Ec-JNK1, which contained a 17.7 kDa TRX-His-S tag and the predicted 44.2 kDa Ec-JNK1 (Figure 2A). IPTG induced the recombinant protein of Ec-JNK1 (lane 3), which was contained in the sediment of bacterial lysate (lane 2). The purified protein of Ec-JNK1 (lane1) was used to produce the antibody. The validation and specificity of mouse anti-EcJNK1 serum was confirmed by western blot with purified protein of Ec-JNK1, host (GS) cells (Figure 2A, lane 5–8) and non-host (FHM) cells successfully translated exogenous JNK1 (Ec-JNK1; Figure 2B). The mouse anti-EcJNK1 serum and pre-immune mouse serum (control), were obtained and used to detect the purified protein of Ec-JNK1 (lane 5, 6) and GS cell lysate (lane 7, 8), respectively. The protein expression levels of Ec-JNK1 were different among three screening cell lines, which translated empty vector pcDNA3.1, wild type (Ec-JNK1), and mutant JNK1(EcJNK1-Δ183-185; Figure 2B, line Ec-JNK1).

### Immunofluorescence staining

**Figure 3:**
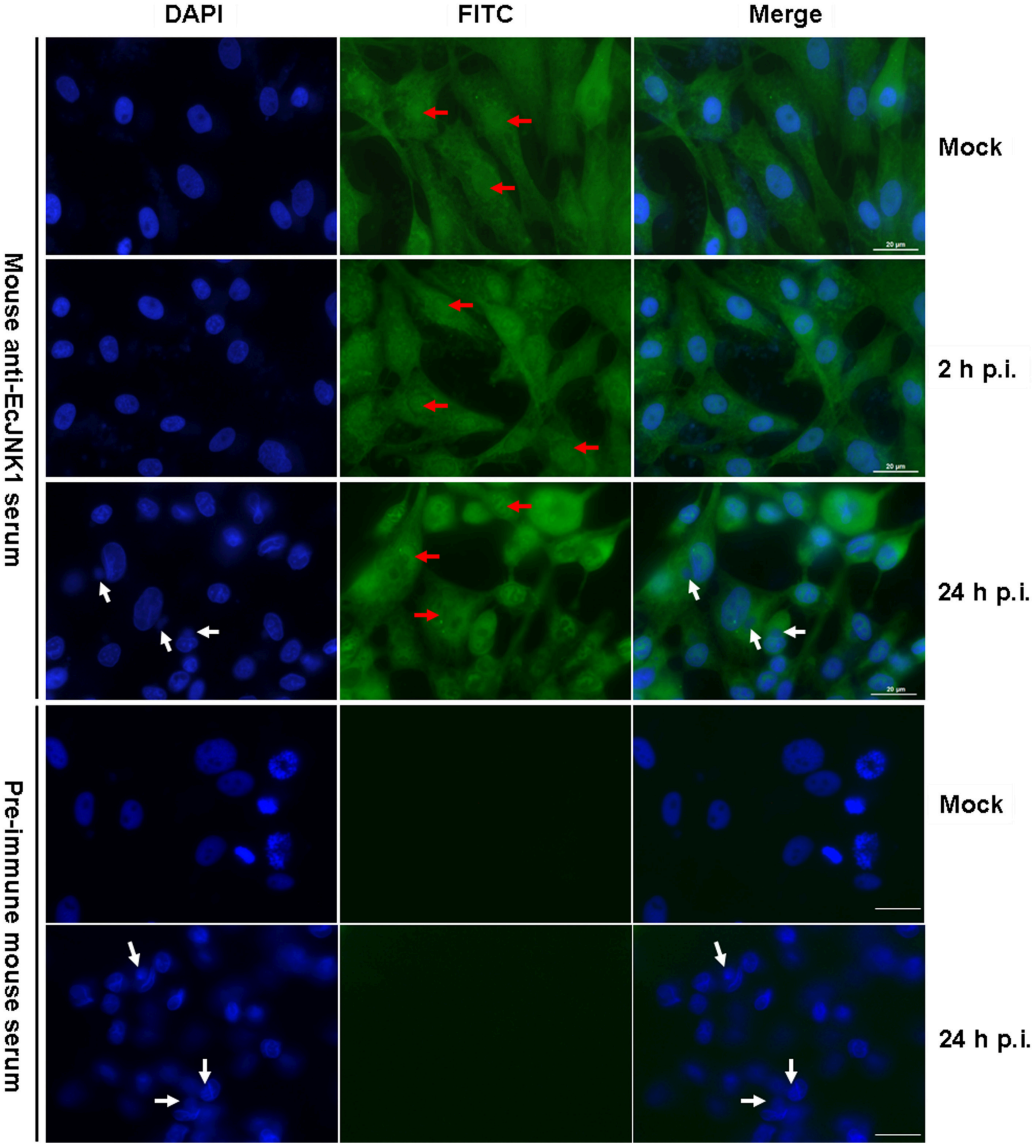
**Intracellular localization of Ec-JNK1 using immunofluorescence staining (bar = 20 μm)**. GS cells were cultured and adhered for 24 h in six-well plates. Immunofluorescence staining was performed to detect the localization of Ec-JNK1 using mouse anti-EcJNK1 serum and Goat anti-mouse IgG-FITC. Pre-immune mouse serum was used as the control. Blue images show the location of the nucleus, stained by DAPI. Red arrows represent the nucleus staining by FITC. White arrows indicate the inclusion body (viral assembly sites).

The subcellular localization and immune responses of Ec-JNK1 were determined by immunofluorescence staining using mouse anti-EcJNK1 serum. In mock- GS cells, Ec-JNK1 was localized in both nucleus and cytoplasm. After infection with SGIV, the localization remained the same situation at indicated times of post-infection. The nucleus (red arrow) of mock and infected cells was positive after testing with the antibody of Ec-JNK1. No protein of Ec-JNK1 was found in the inclusion body (virus assembly sites, white arrow, Huang et al., 2008, 2011a,b). No green fluorescence signal was observed in cells detected by pre-immune mouse serum (Figure 3).

### Activity of Ec-JNK1 on SGIV infection *in vitro*

**Figure 4:**
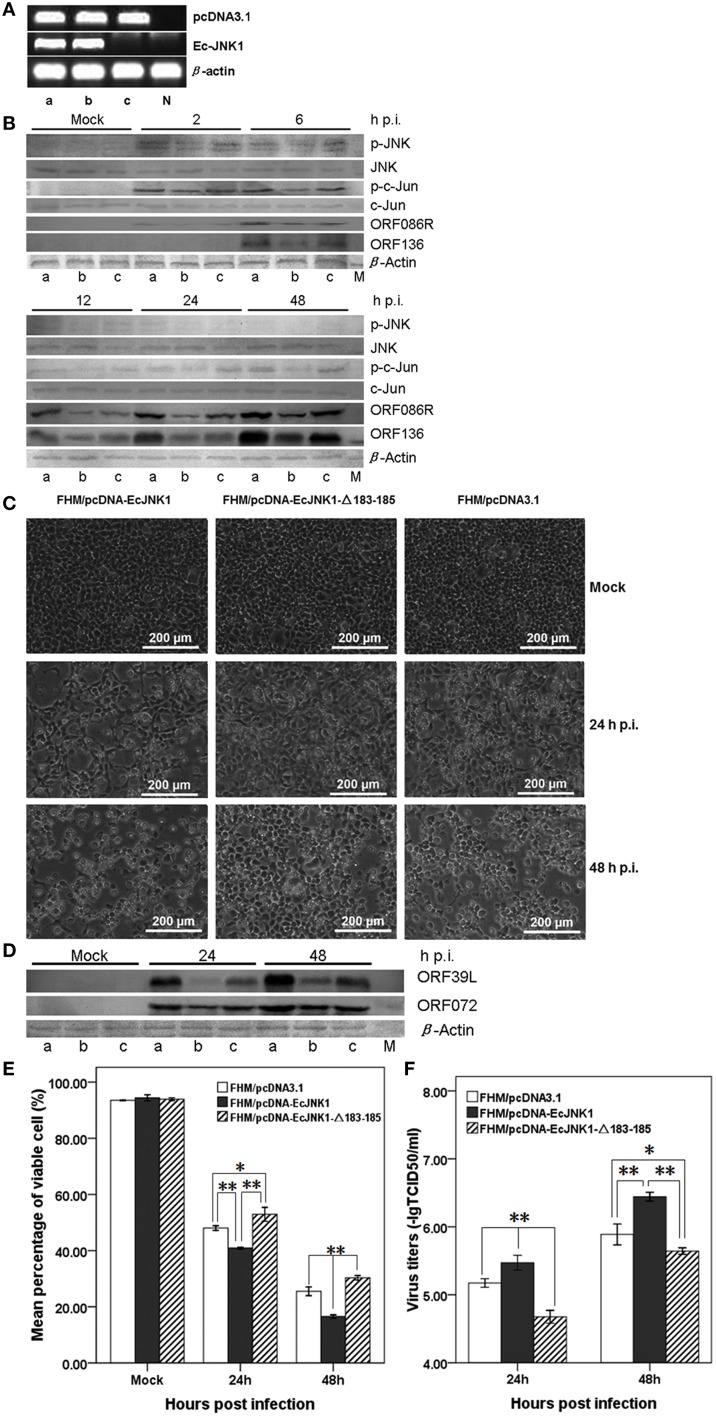
**Activities of Ec-JNK1 and dominant-negative EcJNK1-Δ183-185 mutant on SGIV infection and replication *in vitro***. **(A)** Detection of pcDNA3.1 and Ec-JNK1 mRNA expression in screening FHM cells. Lane a, FHM/pcDNA-EcJNK1; Lane b, FHM/pcDNA-EcJNK1-Δ183-185; Lane c, FHM/pcDNA3.1; N, negative control (FHM). Line pcDNA3.1 and line Ec-JNK1 represented the expression of pcDNA3.1 and Ec-JNK1. Lane a, b, and c represented the same FHM cell throughout this figure. **(B)** The activation of p-JNK and p-c-Jun, protein accumulation of JNK, c-Jun, SGIV immediate-early gene ORF86R, and early gene ORF136 in three cell lines detected by western blot. Compared to the pcDNA3.1 (lane c), Ec-JNK1 (lane a), and dominant-negative EcJNK1-Δ183-185 mutant (lane b) showed opposite effects on the host endogenous JNK and c-Jun phosphorylation and viral protein accumulation of ORF86R and ORF136 during SGIV infection. Total amounts of JNK and c-Jun remained unchanged during SGIV infection. **(C)** Phase microscopy observation of SGIV infection induced CPE in three FHM cell lines above. Ec-JNK1 promoted the process of CPE, while EcJNK1-Δ183-185 mutant inhibited it. **(D)** Accumulation of viral protein SGIV ORF39L and ORF072 in three cell lines above during SGIV infection. EcJNK1-Δ183-185 mutant inhibited the production of viral proteins above (Lane b), while Ec-JNK1 increased it (Lane a). M, Protein Marker. **(E)** Cell viability of SGIV infection in three cell lines above. The viable cells were counted using trypan blue test. **(F)** Comparison of virus titers to SGIV infection in three cell lines. Ec-JNK1 significantly increased the infectivity of SGIV, while dominant-negative Ec-JNK1-Δ183-185 mutant decreased it. Experiments above repeated three times. Data of **(E,F)** represent the means ± S.D. (*n* = 3). ^*^p < 0.05; ^**^p < 0.01.

Method over-expressing dominant-active and/or dominant-negative gene was used to examine the function of Ec-JNK1 *in vitro*. Three FHM cells stably over-expressing pcDNA-EcJNK1, pcDNA-EcJNK1-Δ183-185, and pcDNA3.1 vector (control) (Figure 4A) were screened and cultured to study the roles of Ec-JNK1 on SGIV infection. Wild type and mutant Ec-JNK1 were successfully translated in the screening cell lines (Figure 2B). The high specificity of mouse anti-EcJNK1 serum made the mutant Ec-JNK1 slightly less expression than wild type. To detect the total amounts of JNK (exogenous and endogenous), commercial JNK antibody with multiple species reactivity was used as the primary antibody and detected with chemiluminescent substrates (more sensitive) but not colorimetric reagents. Therefore, the total amounts of JNK were detectable in three cell lines (Figure 4B) and not so changes as Figure 2B. Compared to the control (FHM/pcDNA3.1, Figure 4B, lane c), FHM/pcDNA-EcJNK1 over-expressed the dominant-active Ec-JNK1, promoted the phosphorylation of endogenous JNK and c-Jun, and increased the protein accumulation of SGIV immediate-early gene ORF086R and early gene ORF136 (Figure 4B, lane a). However, the dominant-negative Ec-JNK1-Δ183-185 mutant, partially inhibited the phosphorylation of JNK, showed the opposite effects (Figure 4B, lane b). The altered levels of phosphorylated p-JNK and c-Jun were direct substrates of dominant-active and dominant-negative Ec-JNK1, because total amounts of JNK (exogenous and endogenous) and c-Jun remained unchanged during SGIV infection (Figure 4B). Wild type and mutant JNK1 showed contrary functions on the infection and replication cycle of SGIV. Inhibition of phosphorylation of JNK and c-Jun by EcJNK1-Δ183-185 mutant highly reduced the viral gene transcription and protein expression. At 24 and 48 h p.i., a high amount cytopathic effect (CPE) was observed in FHM/pcDNA-EcJNK1 and FHM/pcDNA3.1 cells, while a few in FHM/EcJNK1-Δ183-185 cells (Figure 4C). Comparing to mock infection, Ec-JNK1 increased the productions of viral structure protein ORF39L and ORF072 (major capsid protein), while the EcJNK1-Δ183-185 mutant decreased them (Figure 4D). Ec-JNK1 significantly decreased the cell viability (40.92% at 24 h p.i. and 16.56% at 48 h p.i.) compared to that of the control (48.02% at 24 h p.i. and 25.50% at 48 h p.i.), while dominant-negative EcJNK1-Δ183-185 mutant significantly increased it (52.93% at 24 h p.i. and 30.33% at 48 h p.i.) during the SGIV infection(Figure 4E). Ec-JNK1 significantly increased the infectivity of SGIV. At 24 and 48 h p.i., the virus titers TCID50 of Ec-JNK1 cell lysates were 6.24 ± 1.04 and 6.31 ± 1.04 times higher than that of the dominant-negative Ec-JNK1-Δ183-185 mutant, and 1.99 ± 1.11 and 3.59 ± 0.82 times higher than that of the control (Figure 4F).

### Effect of Ec-JNK1 on SGIV-induced apoptosis

**Figure 5:**
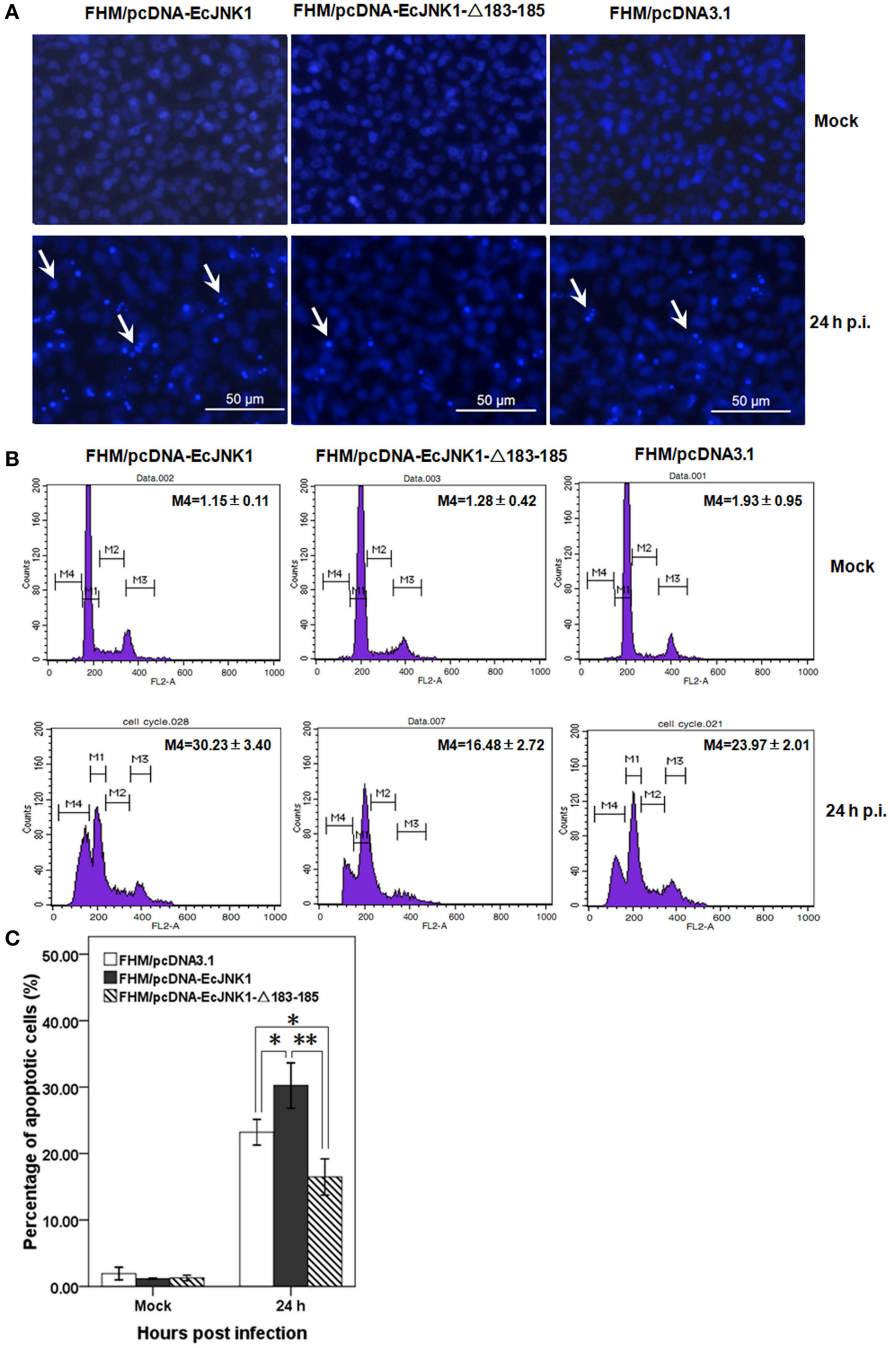
**Roles of Ec-JNK1 and dominant-negative EcJNK1-Δ183-185 mutant on SGIV-induced apoptosis**. **(A)** Cellular nuclear morphology in SGIV infected stable cells. The nucleus were stained with Hoechst 33342 and observed under fluorescence microscopy. Arrows showed the apoptotic bodies. **(B)** Flow cytometry analysis of DNA content in mock- and/or SGIV-infected cells. The quantitative analysis of apoptotic cells in hypodiploid DNA peak (M4, sub-G0/G1 population) was calculated by sub-G0/G1 population/total cell cycle populations and shown in each plot. **(C)** The percentages of apoptotic cells from three independent experiments were indicated on the histogram and statistically analyzed. Error bars indicate S.D. ^*^p < 0.05; ^**^p < 0.01.

SGIV infection induced typical apoptosis in FHM cells. After staining with Hochest, we found that Ec-JNK1 increased the formation of apoptotic bodies compared to that of the control, while dominant-negative EcJNK1-Δ183-185 mutant decreased it (Figure 5A). Prominent sub-G0/G1 peaks (M4), which represented the apoptotic cells, were detected by flow cytometry analyses after infecting with SGIV (Figure 5B). At 24 h p.i., the percentage of apoptotic cells increased significantly in FHM/pcDNA-EcJNK1 (30.23%) compared to that of the control (23.97%), while highly decreased in FHM/pcDNA-Ec-JNK1-Δ183-185 (16.48%; Figure 5C).

**Figure 6:**
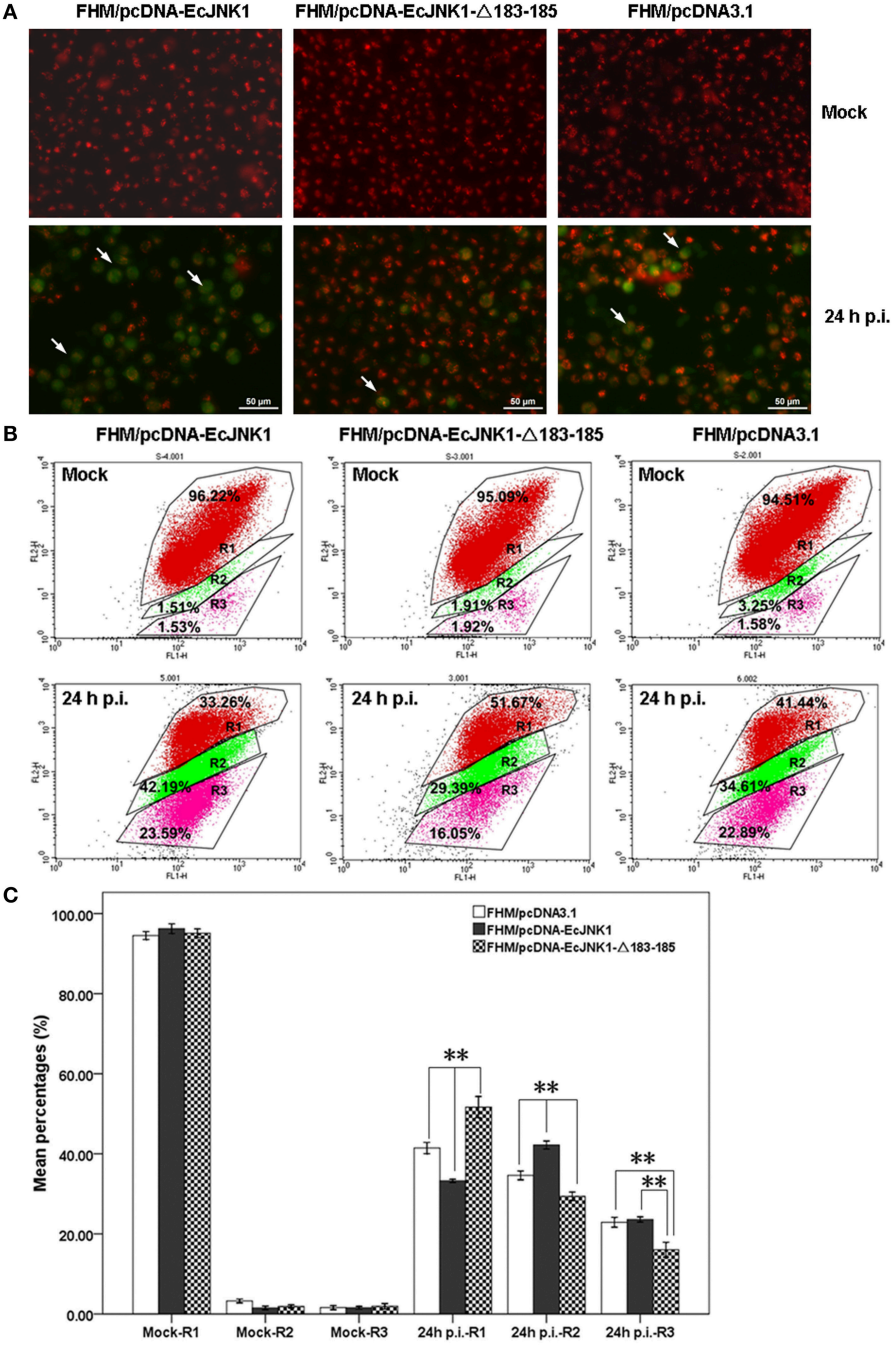
**Different changes of ΔΨm in screening cell lines on SGIV-induced apoptosis**. **(A)** Immunofluorescence microscopy observation of ΔΨm in mock- and/or SGIV-infected cells. Cells with a decreased ΔΨm omitted with green fluorescence (arrowheads). **(B)** Quantitative analysis of cells with depolarized ΔΨm during the SGIV infection by flow cytometry. FL1-H and FL2-H mean the green and red fluorescence by JC-1 staining. R1, healthy cells detected in FL2-H containing red JC-1 aggregates. R2, apoptotic cells detected in FL1-H containing green JC-1 monomers. R3, cells changed from healthy to apoptosis (staining with red and green fluorescence). **(C)** Statistical analysis of fold changes of ΔΨm. R1, R2, and R3 represented the percentage of cells mentioned above. Multiple comparisons of LSD and Dancan in One-Way ANOVA were used to analyze the fold changes of ΔΨm among three cell lines during SGIV infection. ^*^p < 0.05; ^**^p < 0.01.

Mitochondrial function impacts greatly on the cell death and survival on apoptosis. Ec-JNK1 promoted the mitochondrial depolarization during SGIV infection. The dye JC-1 was accumulated in the mitochondria and appeared bright red in mock- cells. At 24 h p.i., the cytoplasm of apoptotic cell with green fluorescence was increased in FHM/pcDNA-EcJNK1 compared to that of the control, but decreased in FHM/pcDNA- EcJNK1-Δ183-185 (Figure 6A). At 24 h p.i., the percentage of cells with depolarized ΔΨm (apoptotic cells) was significantly increased in cells over-expressing Ec-JNK1 (R2 = 42.19%) compared to that of the control (R2 = 34.61%), while decreased in cells expressing the dominant-negative EcJNK1-Δ183-185 mutant (R2 = 29.39%; Figures 6B,C). The percentages of cells containing red JC-1 aggregates (R1) were also significantly different among the three cell lines. So was the cells staining with red and green (R3; Figure 6C).

**Figure 7:**
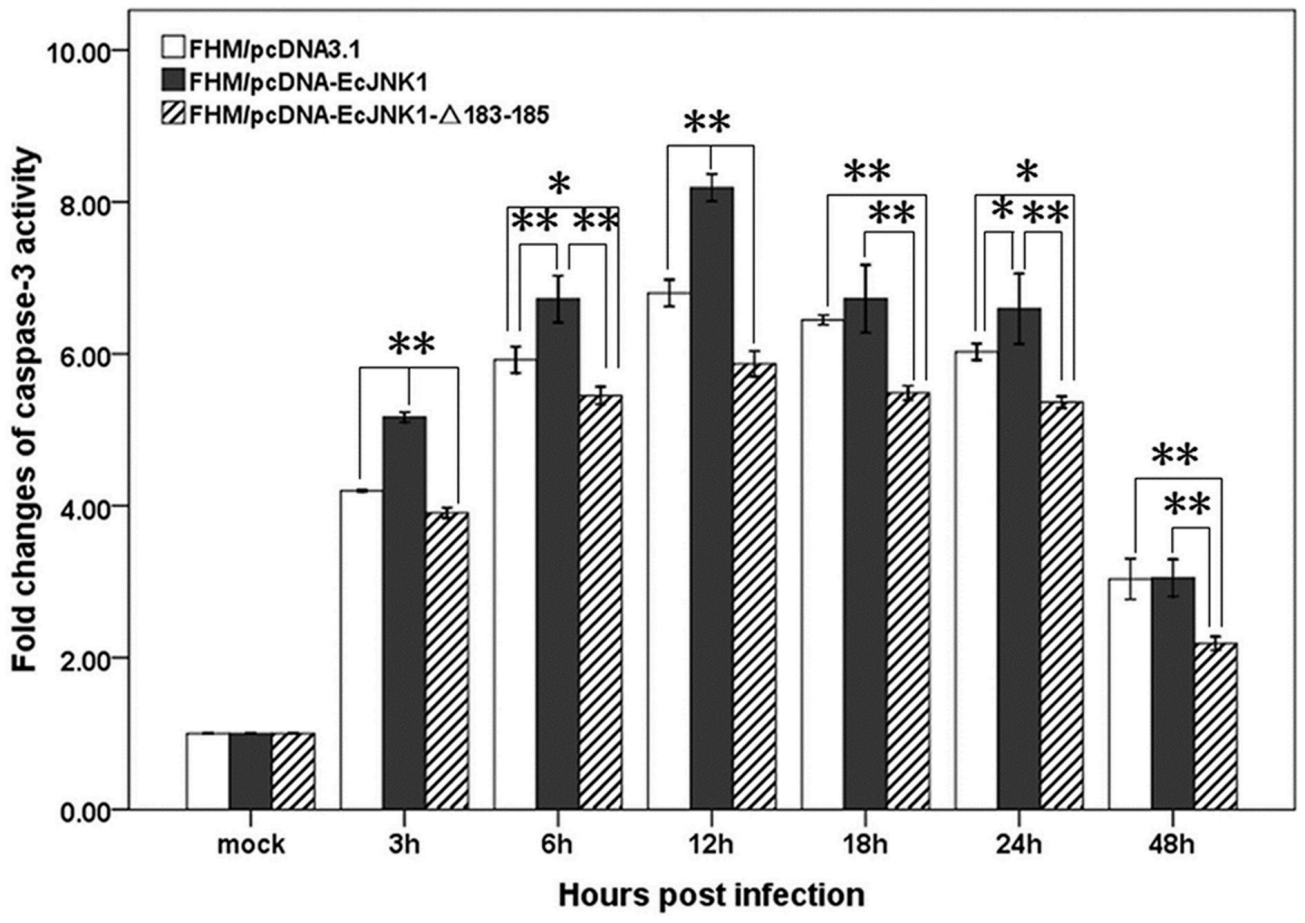
**Measurement of caspase-3 activity in screening cell lines during SGIV infection**. Ec-JNK1 significantly increased the activity of caspase-3 compared to that of the control, while dominant-negative EcJNK1-Δ183-185 mutant decreased it. Fold changes of caspase-3 activity among three cell lines were statistically analyzed using multiple comparisons of LSD and Dancan in One-Way ANOVA. ^*^p < 0.05; ^**^p < 0.01.

Ec-JNK1 increased the activities of caspase-3 in SGIV-induced apoptosis. The activities of caspase-3 in three cell lines were significantly changed and reached a peak level at 12 h p.i. during SGIV infection. The activity of caspase-3 was also significantly different among the three cells from 3 to 48 h p.i. (Figure 7). At 12 h p.i., cells over-expressing Ec-JNK1 significantly enhanced the activity of caspase-3 (fold changes = 8.19 ± 0.18) compared to that of the control (fold changes = 6.80 ± 0.18). However, cells expressing dominant-negative EcJNK1-Δ183-185 mutant (fold changes = 5.87 ± 0.17) highly inhibited it. It indicated that the SGIV-induced apoptosis was caspase-3-dependent. Ec-JNK1 promoted the caspase-3-dependent apoptosis, while dominant-negative mutant inhibited it *in vitro*.

### Activation of reporter genes

**Figure 8:**
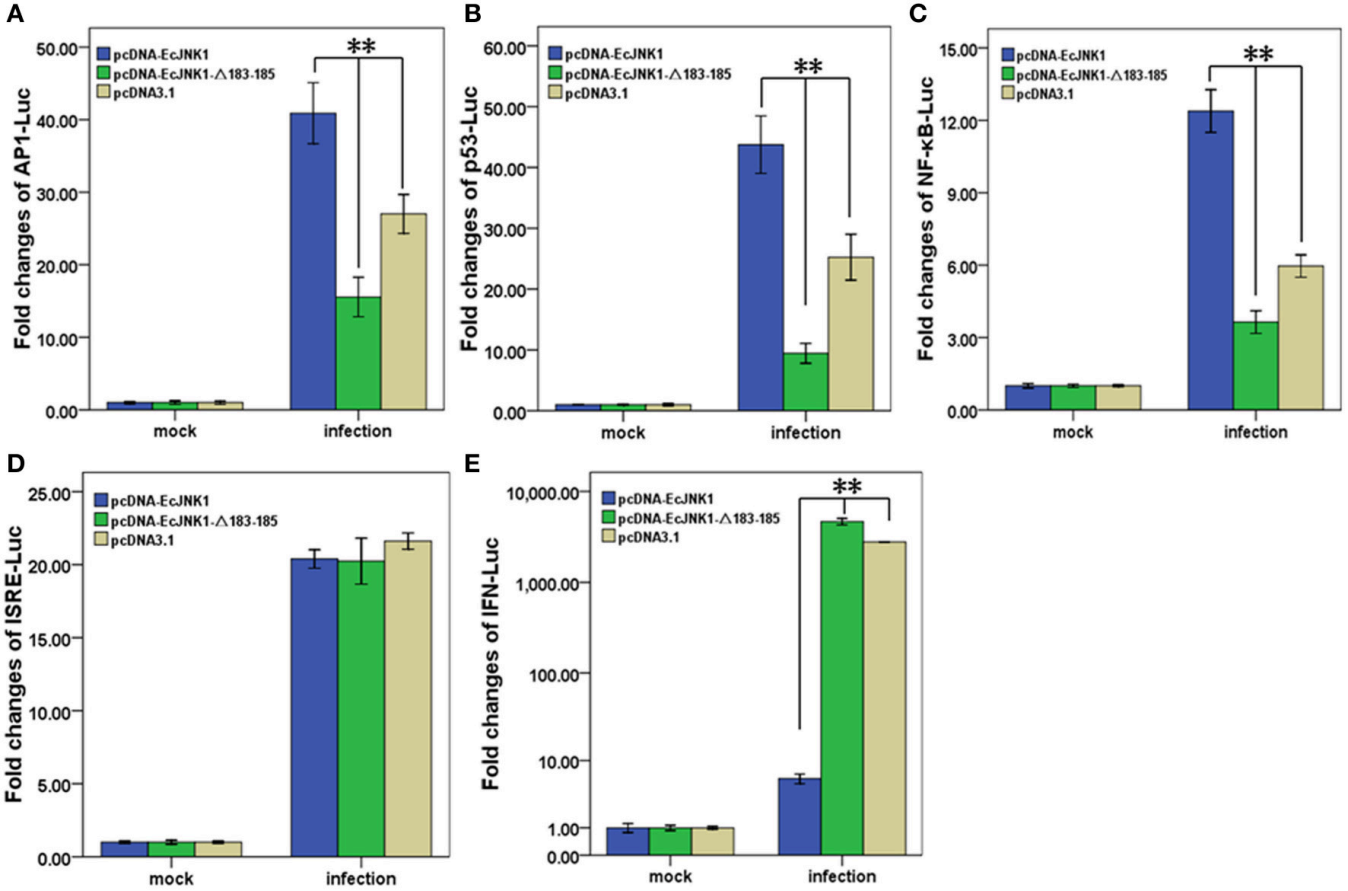
**Activation of reporter genes by Ec-JNK1 and dominant-negative EcJNK1-Δ183-185 mutant during SGIV infection**. Luciferase activities relative to β-galactsidase were obtained in mock- or infected-cells. The quantitative activities of reporter genes modulated by dominant-active Ec-JNK1, dominant-negative EcJNK1-Δ183-185 mutant were measured and statistically analyzed. The empty vector pcDNA3.1 was used as the control. The activities of reporter gene AP1-Luc **(A)**, p53-Luc **(B)**, NF-κB-Luc **(C)**, ISRE-Luc **(D)**, and IFN-Luc **(E)** were showed as fold changes. All data were from three independent experiments and presented as mean ± S.D. (*n* = 3). ^*^p < 0.05; ^**^p < 0.01.

Pathway-specific reporter genes were measure in FHM cell to investigate the role of Ec-JNK1 in cell signal transduction and SGIV infection. All examined reporter genes were significantly activated after infecting with SGIV. At 48 h p.i., the activities of AP1-Luc, p53-Luc and NF-κB-Luc were significantly (*p* < 0.01) increased in cells transfected with pcDNA-EcJNK1 (fold changes = 40.89 ± 4.20, 43.75 ± 4.72, and 12.38 ± 0.89) compared to that of the control pcDNA3.1 (fold changes = 27.02 ± 2.68, 25.25 ± 3.77, and 5.96 ± 0.46), but significantly decreased (*p* < 0.01) in cells transfected with pcDNA-EcJNK1-Δ183-185 (fold changes = 15.56 ± 2.72, 9.44 ± 1.64, and 3.64 ± 0.47) (Figures 8A–C). However, the activities of IFN-Luc were opposite. Compared to that of pcDNA3.1 (fold changes = 2783.61 ± 26.85), Ec-JNK1 significantly inhibited (*p* < 0.01) the activation of IFN-Luc (fold changes = 5.96 ± 0.83), while EcJNK1-Δ183-185 mutant promoted it (fold changes = 4673.11 ± 382.09) (Figure 8E). The activities of ISRE-Luc showed no significant differences among cells transfected with pcDNA-EcJNK1, pcDNA-EcJNK1-Δ183-185 and/or pcDNA3.1 (Figure 8D).

## Discussion

There are three genes, JNK1, JNK2, and JNK3 in the JNK family which all contain TPY in their activation motif (Kyriakis et al., 1994). In this study, a JNK1, Ec-JNK1, was obtained and characterized. It contains a TPY motif in the multi-domains S_TKc. Phylogenetic analysis suggests that JNK1 is evolutionarily conserved. The expressions of JNK1 and JNK2 have been found to be ubiquitous, while the expression of JNK3 is brain-specific (Davis, 2000; Zhang and Dong, 2007). JNK1 involves in innate immune (Rincón et al., 2001; Arbour et al., 2002; Chen et al., 2007). Ec-JNK1 was found to distribute in all examined immune tissues and showed pathogenic-dependent immune response in juvenile grouper. The different reaction time of JNK1 in individual and/or cell could be related with infecting time of SGIV. SGIV injected in muscle of grouper needs time to reach and infect cells of spleen and liver, while direct infection of SGIV in FHM cell activates JNK1 early after infection (more quickly). Phosphorylation and activation of JNKs has crucial impacts on the viral infection (Nacken et al., 2014; Berard et al., 2015). The localization of Ec-JNK1 showed no changes during SGIV infection. However, phosphorylation of JNK (2 h p.i.) and c-Jun (24 h p.i.) was activated and respectively located in the nuclear and inclusion body of SGIV-infected cell, and no changes were found in the total amounts of JNK (Huang et al., 2011a). These data demonstrate that phosphorylation of JNK1 could be very important in SGIV infection. Therefore, ectopically expression of dominant-active (Ec-JNK1) and dominant-negative Ec-JNK1 (EcJNK1-Δ183-185 mutant, which partially inhibited the phosphorylation of Ec-JNK1) were employed to study its functions in SGIV infection.

JNK molecules are required for virus entry and gene transfer (Lee et al., 2011) and replication (McLean and Bachenheimer, 1999; Pan et al., 2006). Similar to EAGS cells (Huang et al., 2011a), phosphorylation JNK was spiked at early stage of SGIV infection in FHM cell. Amplification of JNK signaling is necessary for completing the viral replication of murine gammaerpesvirus cycle (Stahl et al., 2012). Ec-JNK1 was found to promote the SGIV infection and replication and the phosphorylation of c-Jun, while the dominant-negative EcJNK1-Δ183-185 mutant showed the opposite effects. The dual phosphorylation motif TPY of JNK is important for transferring signals from the cell membrane to nuclear transcription factors, by increasing their ability to activate transcription factors (Kyriakis et al., 1994; Dong et al., 2002; Stahl et al., 2012). Virus seems to activate the JNK-AP-1 pathway to promote infection (Kumar et al., 1998; Holloway and Coulson, 2006; Berard et al., 2015). The JNK signal pathway represents a major physiological mechanism of regulation of c-Jun that relates to AP1 (Gupta et al., 1996; Davis, 2000). Mediation through c-Jun/AP1 was found in the mechanisms involved in JNK-mediated virus replication (Stahl et al., 2012; Berard et al., 2015) and/or virus-induced cell death (Yin et al., 2012). The SGIV infection activated AP-1 (Huang et al., 2011a), and the activation of AP-1 was found to require activation of JNK1 and c-Jun. In addition, activation of Ec-JNK1 was triggered by SGIV initially entry (peaked at about 2 h). Chemical inhibitor SP600125 (Huang et al., 2011a) or dominant-negative EcJNK1-Δ183-185 mutant significantly reduced but did not eliminate SGIV entry and replication, which likes the lentivirus, a “positive cooperativity” mechanism might be at work (Lee et al., 2011).

To efficiently infect and replicate, SGIV modulated JNK1 to inactivate the antiviral signaling, enhance the SGIV-induced apoptosis and activate transcription factors. Viruses have evolved elaborate strategies to evade detection and destruction by host immune responses, including targeting pathways for antigen presentation, programmed cell death, and cytokine- and chemokine-mediated signaling (Tortorella et al., 2000; Ciccaglione et al., 2007). Virus can modulate and/or interfere with host innate immunity by inactivating antiviral gene and signaling, such as interferon (IFN; Hengel et al., 2005; Ambrose and Mackenzie, 2011; Taylor and Mossman, 2013). SGIV activated Ec-JNK1 to inhibit the production of type I IFN independent of ISRE activation. Virus-induced apoptosis follows a pro-apoptosis pathway involving the activation of JNK (Clarke et al., 2004; Beckham et al., 2007), NF-κB (Santoro et al., 2003; Hansberger et al., 2007), and p53 (Gougeon and Piacentini, 2009). To facilitate the viral propagation, SGIV enhanced the typical apoptosis by activating Ec-JNK1. Phosphorylation of JNK1 by SGIV promoted the production of SGIV ORF136, which could contribute to virus transmission and SGIV-induced apoptosis (Huang et al., 2008). Hochest, PI, and JC-1 staining analyses showed that Ec-JNK1 significantly promoted the SGIV-induced apoptosis, while dominant-negative EcJNK1-▵183-185 mutant suppressed it. Ec-JNK1 increased the activation of caspase 3 suggesting a positive relationship of caspase-3-dependent apoptosis and Ec-JNK1 during SGIV infection. Ec-JNK1 could enhance SGIV-induced apoptosis by activating reporter gene NF-κB and p53. Noncanonical NF-κB pathway was stimulated along with viral infections and had a vital role in negatively regulating type I IFN induction (Jin et al., 2014). Further investigations need to be done as to how p53 signaling activation with SGIV infection and the cross talk of JNK1 and investigated signal pathways above.

JNK molecules were activated to involve in different stages of replication cycles for many DNA virus (Lee et al., 2011; Stahl et al., 2012; Klein et al., 2015) and RNA virus (Si et al., 2005; Tung et al., 2010; Wei et al., 2011). The JNK signaling pathway has been confirmed to be involved in the infection of iridovirus (Chitnis et al., 2008; Huang et al., 2011a), which are large DNA viruses isolated from invertebrate and poikilothermic vertebrates (Williams et al., 2005). SGIV is a *Ranavirus* belonging to the genera *Iridovirus* of family Iridoviridae. JNK1 regulation of viral replication could act as specific steps of the viral replication cycle, including entry, gene transcription, protein expression, and assembly. Phosphorylation of JNK and c-Jun could be detected from the viral entry of SGIV (1 and 0.5 h p.i., respectively; Huang et al., 2011a). Again, our data showed that the phosphorylation peaked at the early stage of SGIV infection. These findings suggest that JNK1 and c-Jun activation occurs early after infection and is functionally amplified as productive viral replication proceeds to facilitate viral gene expression for completing the replication cycle. Similar to the lytic replication cycle of herpesvirus (Sharma-Walia et al., 2005; Pan et al., 2006; Stahl et al., 2012), JNK1 and/or c-Jun phosphorylation enhanced SGIV immediate-early gene ORF086R and early gene ORF136 expressions. However, inhibition of phosphorylation of JNK and c-Jun by JNK1 mutant did not globally block the expression of viral genes contribute to the formation of virus factories and assembly, including the iridovirus ORF086R (Xia et al., 2009), structure protein ORF072 (Ou-yang et al., 2012b) and ORF39L (Zhang et al., 2015). These data demonstrate that JNK1 is also essential to the late stage in the replication process of iridoviruses. Further studies of direct interaction of JNK1 with SGIV protein need to be done to delineate the molecular mechanism of JNK activation by SGIV infection.

In conclusion, Ec-JNK1 has been involved in the immune response to pathogen challenges *in vivo* and the SGIV infection *in vitro*. Previous studies indicated the importance of JNK1/2 signaling pathway in iridovirius replication (Chitnis et al., 2008; Huang et al., 2011a). Our findings define novel functions of JNK1 molecule on irdovirus infection in fish and reveal the molecular mechanism mediated by JNK1 in evasion and infection of iridovirus. Activation of JNK1 is required for the evasion and replication of iridovirus. Opposite effects of wild type and mutant JNK1 on production of viral protein showed that JNK1 involved in replication cycle of iridovirus. Many mammalian viruses directly regulated NF-κB, p53, and IFN signal pathway for evasion and infection (Santoro et al., 2003; Kinpara et al., 2013; Taylor et al., 2013). However, iridovirus modulated these signal pathways by activating JNK1. A “positive cooperativity” mechanism mediated by JNK1 could be at work for the efficient infection and replication of iridovirus. To facilitate the viral entry and replication, iridovirus SGIV phosphyrylated JNK1 which promoted the activation of transcription factors c-Jun, AP-1, NF-κB, and p53. SGIV usurped JNK1 as infection processes to block the type I IFN signaling and promote the caspase-3-dependent apoptosis for efficient replication. The novel functions of JNK1 on viral evasion and replication, and virus-induced apoptosis will contribute to the molecular mechanism of iridovirus pathogenesis.

## Author contributions

All authors of this manuscript made contributions as following: the conception or design of the work, acquisition, analysis, interpretation of data for the work, and drafting the work or revising it critically for important intellectual content, and final approval of the version to be published, and agreement to be accountable for all aspects of the work in ensuring that questions related to the accuracy or integrity of any part of the work are appropriately investigated and resolved. Each author's main contribution is different. MG conceived and designed the experiments, performed the experiments, analyzed the data, wrote the paper, prepared figures, and/or tables. JW analyzed the data, contributed reagents/materials/analysis tools, and revising the work critically for important intellectual content. XH contributed acquisition and interpretation of data for the work. YZ contributed materials for the work and reviewed drafts of the paper. YY contributed interpretation of data for the work and reviewed drafts of the paper. QQ conceived and designed the experiments, reviewed drafts of the paper, provided final approval of the version to be published.

### Conflict of interest statement

The authors declare that the research was conducted in the absence of any commercial or financial relationships that could be construed as a potential conflict of interest.
