# Supplementary material for: JNK1 Derived from Orange-Spotted Grouper, Epinephelus coioides, Involving in the Evasion and Infection of Singapore Grouper Iridovirus (SGIV)
Source: Front Microbiol. 2016 Feb 10;7:121. doi: 10.3389/fmicb.2016.00121 (PMC4748057; doi:10.3389/fmicb.2016.00121)
Supplement: Supplementary file 1 [file Image1.PDF]

1 ACGCGGGGAAGGTTTCGACGACGTTGGTATGAAATGGCAGAAACTGCTTGTGTCACGGAAGCACAAAGATTAGGTTACGT

81 AGAAGCTACACATGTATTCTGCGATGATCCCGGCCTGCTAGTGTGAGGGAAACGGGAGTGTATCCGGAGGAAAAAGGGAT

161 TAAAGAGCTGTTTTTCTCCTTTTAAAAAAAGAGAAACGTCCGGGTTGTGTCTCCAGCAGGCAGCAACGGCGACTGCGGGT

241 CGACACTGGTGGAATTAACCGGAGAGGGGTCTTGCGAGCTCCACTTGAATGCCCAGCCATAGCGTTCGTCCAAGGCTTCA

321 CC**ATG**AACCGGAACAAGCGTGAAAAGGAGTACTACAGTATAGATGTGGGCGATTCGACTTTTATGGTTTTAAAGCGCTAC

M N R N K R E K E Y Y S I D V G D S T F M V L K R Y

401 CAGAACCTCAGACCCATCGGATCCGGAGCACAGGGAATTGTCTGTTCAGCGTATGACCACAACTTGGAGAGGAACGTCGC

Q N L R P I G S G A Q G I V C S A Y D H N L E R N V A

481 CATCAAGAAGCTGAGCCGGCCGTTTCAGAATCAAACTCATGCGAAACGGGCTTACAGGGAACTGGTGCTAATGAAATGTG

I K K L S R P F Q N Q T H A K R A Y R E L V L M K C V

561 TCAACCACAAGAACATAATCGGCCTTTTAAATGTATTCACACCACAGAAGACACTGGAAGAATTCCAAGATGTGTATCTG

N H K N I I G L L N V F T P Q K T L E E F Q D V Y L

641 GTGATGGAGCTGATGGATGCCAACCTCTGCCAGGTGATTCAGATGGAGCTGGACCACGAGAGGCTGTCCTACCTGCTCTA

V M E L M D A N L C Q V I Q M E L D H E R L S Y L L Y

721 CCAGATGCTGTGTGGAATCAAACACCTGCACGCTGCTGGCATCATACACAGGGACCTGAAGCCGAGCAACATCGTGGTGA

Q M L C G I K H L H A A G I I H R D L K P S N I V V K

801 AGTCTGACTGCACACTGAAGATCCTGGACTTTGGCTTGGCCAGGACAGCTGCCACCGGCCTCCTCATGACGCCCTACGTG

S D C T L K I L D F G L A R T A A T G L L M T P Y V

881 GTCACCCGCTACTACCGCGCCCCAGAGGTCATCCTGGGCATGGGCTACCAGGCCAACGTTGATGTCTGGTCTGTTGGCTG

V T R Y Y R A P E V I L G M G Y Q A N V D V W S V G C

961 CATCATGGCTGAAATGGTCCGGGGTAGTGTGTTGTTTCCAGGCACCGATCATATCGACCAGTGGAATAAGGTGATCGAGC

I M A E M V R G S V L F P G T D H I D Q W N K V I E Q

1041 AGCTGGGGACACCGTCTCAGGAGTTCCTGATGAAGCTCAACCAGTCGGTGAGGACCTACGTGGAGAACAGGCCACGGTAT

L G T P S Q E F L M K L N Q S V R T Y V E N R P R Y

1121 GCGGGCTACAGCTTTGAGAAGCTCTTCCCTGATGTCCTGTTCCCTGCAGACTCTGAACACAACAAACTGAAAGCGAGCCA

A G Y S F E K L F P D V L F P A D S E H N K L K A S Q

1201 AGCTCGAGACCTACTATCCAAGATGCTGGTAATAGACGCTTCAAAGCGGATCTCAGTGGACGAGGCTCTCCAGCACCCTT

A R D L L S K M L V I D A S K R I S V D E A L Q H P Y

1281 ATATCAACGTGTGGTACGACCCGACTGAAGTGGAGGCGCCACCACCCGCGATCACAGACAAGCAGCTGGATGAAAGAGAG

I N V W Y D P T E V E A P P P A I T D K Q L D E R E

1361 CACACAGTGGAGGAGTGGAAAGAGTTGATATACAAAGAAGTGTTGGACTGGGAAGAAAGGACAAAGAACGGTGTCATCAG

H T V E E W K E L I Y K E V L D W E E R T K N G V I R

1441 GGGACAGTCAGCGTCCATAGCACAGGTGCAACAG**TGA**GCAGCAGCCCCTAGCAGCACCACCAGCACCACACCTCCATGTC

G Q S A S I A Q V Q Q *

1521 CTCCTCCACCTCTGTCAACGATGCTCCTCCATGTCCACCGACCCATCCCTGACCGACACTGACAGCAGCCTGGAGATGGC

1601 CAGCGCCGCCGCCGCTGCTGCTGCTACAGCCACCGGCCCCCTGGGCTGCTGCTACAGCAACCAGCCCCCTGGGCTGCTGC

1681 AGATGACTATCATCTACACCTCCTCCTGGTCCTGGTCCTGGTCCTGGGCTGGGCCTCGCCCCCTGGTCACATCCAAGACC

1761 ACTAGCCCATCATTGACAGAGGTGGAGGAGGAC

FIGURE S1. Nucleotide and deduced amino acid sequences of Ec-JNK1. The predicted phosphorylation motif “TPY” (red) and serine/threonine protein kinase (S_TKc) domains were boxed. The start codon (ATG) and stop codon (TGA) were bold.
