# Supplementary material for: JNK1 Derived from Orange-Spotted Grouper, Epinephelus coioides, Involving in the Evasion and Infection of Singapore Grouper Iridovirus (SGIV)
Source: Front Microbiol. 2016 Feb 10;7:121. doi: 10.3389/fmicb.2016.00121 (PMC4748057; doi:10.3389/fmicb.2016.00121)
Supplement: Supplementary file 2 [file Image2.pdf]

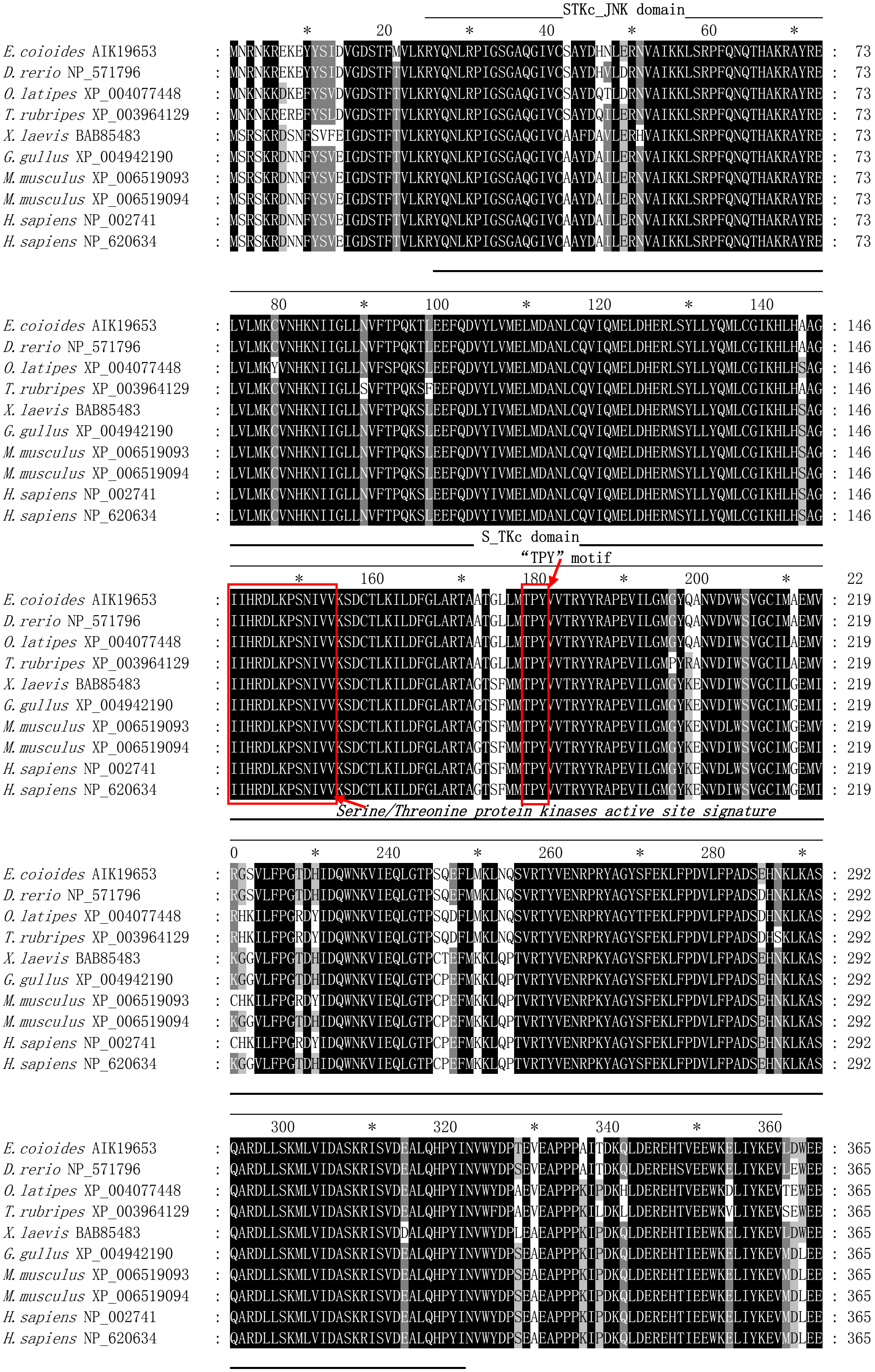
STKc_JNK domain
* 20 * 40 * 60 *
E. coioides AIK19653 : MNRNKREKEYYSIDVGDSTFMVLKRYQNLRPIGSGAQGIVCSAYDHNLERNVAIKKLSRPFQNQTHAKRAYRE : 73
D. rerio NP_571796 : MNRNKREKEYYSIDVGDSTFTVLKRYQNLRPIGSGAQGIVCSAYDHVLDRNVAIKKLSRPFQNQTHAKRAYRE : 73
O. latipes XP_004077448 : MNKNKKDKEFYSVDVGDSTFTVLKRYQNLRPIGSGAQGIVCSAYDQTLDRNVAIKKLSRPFQNQTHAKRAYRE : 73
T. rubripes XP_003964129 : MNKNKREREFYSLDVGDSTFTVLKRYQNLRPIGSGAQGIVCSAYDQILERNVAIKKLSRPFQNQTHAKRAYRE : 73
X. laevis BAB85483 : MSRSKRDSNFSVFEIGDSTFTVLKRYQNLKPIGSGAQGIVCAAFDAVLERHVAIKKLSRPFQNQTHAKRAYRE : 73
G. gullus XP_004942190 : MSRSKRDNNFYSVEIGDSTFTVLKRYQNLKPIGSGAQGIVCAAYDAILERNVAIKKLSRPFQNQTHAKRAYRE : 73
M. musculus XP_006519093 : MSRSKRDNNFYSVEIGDSTFTVLKRYQNLKPIGSGAQGIVCAAYDAILERNVAIKKLSRPFQNQTHAKRAYRE : 73
M. musculus XP_006519094 : MSRSKRDNNFYSVEIGDSTFTVLKRYQNLKPIGSGAQGIVCAAYDAILERNVAIKKLSRPFQNQTHAKRAYRE : 73
H. sapiens NP_002741 : MSRSKRDNNFYSVEIGDSTFTVLKRYQNLKPIGSGAQGIVCAAYDAILERNVAIKKLSRPFQNQTHAKRAYRE : 73
H. sapiens NP_620634 : MSRSKRDNNFYSVEIGDSTFTVLKRYQNLKPIGSGAQGIVCAAYDAILERNVAIKKLSRPFQNQTHAKRAYRE : 73
80 * 100 * 120 * 140
E. coioides AIK19653 : LVLMKCVNHKNIIGLLNVFTPQKTLEEFQDVYLVMELMDANLCQVIQMELDHERLSYLLYQMLCGIKHLHAAG : 146
D. rerio NP_571796 : LVLMKCVNHKNIIGLLNVFTPQKTLEEFQDVYLVMELMDANLCQVIQMELDHERLSYLLYQMLCGIKHLHAAG : 146
O. latipes XP_004077448 : LVLMKYVNHKNIIGLLNVFSPQKSLEEFQDVYLVMELMDANLCQVIQMELDHERLSYLLYQMLCGIKHLHSAG : 146
T. rubripes XP_003964129 : LVLMKCVNHKNIIGLLSVFTPQKSFEEFQDVYLVMELMDANLCQVIQMELDHERLSYLLYQMLCGIKHLHAAG : 146
X. laevis BAB85483 : LVLMKCVNHKNIIGLLNVFTPQKSLEEFQDLYIVMELMDANLCQVIQMELDHERMSYLLYQMLCGIKHLHSAG : 146
G. gullus XP_004942190 : LVLMKCVNHKNIIGLLNVFTPQKSLEEFQDVYIVMELMDANLCQVIQMELDHERMSYLLYQMLCGIKHLHSAG : 146
M. musculus XP_006519093 : LVLMKCVNHKNIIGLLNVFTPQKSLEEFQDVYIVMELMDANLCQVIQMELDHERMSYLLYQMLCGIKHLHSAG : 146
M. musculus XP_006519094 : LVLMKCVNHKNIIGLLNVFTPQKSLEEFQDVYIVMELMDANLCQVIQMELDHERMSYLLYQMLCGIKHLHSAG : 146
H. sapiens NP_002741 : LVLMKCVNHKNIIGLLNVFTPQKSLEEFQDVYIVMELMDANLCQVIQMELDHERMSYLLYQMLCGIKHLHSAG : 146
H. sapiens NP_620634 : LVLMKCVNHKNIIGLLNVFTPQKSLEEFQDVYIVMELMDANLCQVIQMELDHERMSYLLYQMLCGIKHLHSAG : 146
S_TKc domain
“TPY” motif
* 160 * 180 * 200 * 22
E. coioides AIK19653 : IIHRDLKPSNIVVKSDCTLKILDFGLARTAATGLLMTPYVVTRYYRAPEVILGMGYQANVDVWSVGCIMAEMV : 219
D. rerio NP_571796 : IIHRDLKPSNIVVKSDCTLKILDFGLARTAATGLLMTPYVVTRYYRAPEVILGMGYQANVDVWSIGCIMAEMV : 219
O. latipes XP_004077448 : IIHRDLKPSNIVVKSDCTLKILDFGLARTAATGLLMTPYVVTRYYRAPEVILGMGYQANVDIWSVGCILAEMV : 219
T. rubripes XP_003964129 : IIHRDLKPSNIVVKSDCTLKILDFGLARTAATGLLMTPYVVTRYYRAPEVILGMPYRANVDIWSVGCILAEMV : 219
X. laevis BAB85483 : IIHRDLKPSNIVVKSDCTLKILDFGLARTAGTSFMMTPYVVTRYYRAPEVILGMGYKENVDIWSVGCILGEMI : 219
G. gullus XP_004942190 : IIHRDLKPSNIVVKSDCTLKILDFGLARTAGTSFMMTPYVVTRYYRAPEVILGMGYKENVDIWSVGCIMGEMI : 219
M. musculus XP_006519093 : IIHRDLKPSNIVVKSDCTLKILDFGLARTAGTSFMMTPYVVTRYYRAPEVILGMGYKENVDLWSVGCIMGEMV : 219
M. musculus XP_006519094 : IIHRDLKPSNIVVKSDCTLKILDFGLARTAGTSFMMTPYVVTRYYRAPEVILGMGYKENVDIWSVGCIMGEMI : 219
H. sapiens NP_002741 : IIHRDLKPSNIVVKSDCTLKILDFGLARTAGTSFMMTPYVVTRYYRAPEVILGMGYKENVDLWSVGCIMGEMV : 219
H. sapiens NP_620634 : IIHRDLKPSNIVVKSDCTLKILDFGLARTAGTSFMMTPYVVTRYYRAPEVILGMGYKENVDIWSVGCIMGEMI : 219
Serine/Threonine protein kinases active site signature
0 * 240 * 260 * 280 *
E. coioides AIK19653 : RGSVLFPGTDHIDQWNKVIEQLGTPSQEFLMKLNQSVRTYVENRPRYAGYSFEKLFPDVLFPADSEHNKLKAS : 292
D. rerio NP_571796 : RGSVLFPGTDHIDQWNKVIEQLGTPSQEFMMKLNQSVRTYVENRPRYAGYSFEKLFPDVLFPADSDHNKLKAS : 292
O. latipes XP_004077448 : RHKILFPGRDYIDQWNKVIEQLGTPSQDFLMKLNQSVRTYVENRPRYAGYTFEKLFPDVLFPADSDHNKLKAS : 292
T. rubripes XP_003964129 : RHKILFPGRDYIDQWNKVIEQLGTPSQDFLMKLNQSVRTYVENRPRYAGYSFEKLFPDVLFPADSDHSKLKAS : 292
X. laevis BAB85483 : KGGVLFPGTDHIDQWNKVIEQLGTPCTEFMKKLQPTVRTYVENRPKYAGYSFEKLFPDVLFPADSEHNKLKAS : 292
G. gullus XP_004942190 : KGGVLFPGTDHIDQWNKVIEQLGTPCPEFMKKLQPTVRTYVENRPKYAGYSFEKLFPDVLFPADSEHNKLKAS : 292
M. musculus XP_006519093 : CHKILFPGRDYIDQWNKVIEQLGTPCPEFMKKLQPTVRTYVENRPKYAGYSFEKLFPDVLFPADSEHNKLKAS : 292
M. musculus XP_006519094 : KGGVLFPGTDHIDQWNKVIEQLGTPCPEFMKKLQPTVRTYVENRPKYAGYSFEKLFPDVLFPADSEHNKLKAS : 292
H. sapiens NP_002741 : CHKILFPGRDYIDQWNKVIEQLGTPCPEFMKKLQPTVRTYVENRPKYAGYSFEKLFPDVLFPADSEHNKLKAS : 292
H. sapiens NP_620634 : KGGVLFPGTDHIDQWNKVIEQLGTPCPEFMKKLQPTVRTYVENRPKYAGYSFEKLFPDVLFPADSEHNKLKAS : 292
300 * 320 * 340 * 360
E. coioides AIK19653 : QARDLLSKMLVIDASKRISVDEALQHPYINVWYDPTEVEAPPPAITDKQLDEREHTVEEWKELIYKEVLDWEE : 365
D. rerio NP_571796 : QARDLLSKMLVIDASKRISVDEALQHPYINVWYDPSEVEAPPPAITDKQLDEREHSVEEWKELIYKEVLEWEE : 365
O. latipes XP_004077448 : QARDLLSKMLVIDASKRISVDEALQHPYINVWYDPAEVEAPPPKIPDKHLDEREHTVEEWKDLIYKEVTEWEE : 365
T. rubripes XP_003964129 : QARDLLSKMLVIDASKRISVDEALQHPYINVWFDPAEVEAPPPKILDKLLDEREHTVEEWKVLIYKEVSEWEE : 365
X. laevis BAB85483 : QARDLLSKMLVIDASKRISVDDALQHPYINVWYDPLEAEAPPPKIPDKQLDEREHTIEEWKELIYKEVLDWEE : 365
G. gullus XP_004942190 : QARDLLSKMLVIDASKRISVDEALQHPYINVWYDPSEAEAPPPKIPDKQLDEREHTIEEWKELIYKEVMDLEE : 365
M. musculus XP_006519093 : QARDLLSKMLVIDASKRISVDEALQHPYINVWYDPSEAEAPPPKIPDKQLDEREHTIEEWKELIYKEVMDLEE : 365
M. musculus XP_006519094 : QARDLLSKMLVIDASKRISVDEALQHPYINVWYDPSEAEAPPPKIPDKQLDEREHTIEEWKELIYKEVMDLEE : 365
H. sapiens NP_002741 : QARDLLSKMLVIDASKRISVDEALQHPYINVWYDPSEAEAPPPKIPDKQLDEREHTIEEWKELIYKEVMDLEE : 365
H. sapiens NP_620634 : QARDLLSKMLVIDASKRISVDEALQHPYINVWYDPSEAEAPPPKIPDKQLDEREHTIEEWKELIYKEVMDLEE : 365

```
                                       *      380         *      400         *      420         *
E.coioides AIK19653          : RTKNGVIRGQ------------------SASIAQVQQ------------------------------------ : 384
D.rerio NP_571796            : RTKNGVIRGQPASL--AVS-SDSHEPSTSSSSINDVSSMSTEVT-------LTSDTDSSQE--TSNGALHCCR : 426
O.latipes XP_004077448       : WTKNGVIKGQPPPLGAAVIDSPPQPTSSASSSTNDVSSMSTEPSDPSSDPTMASETDSSLNGHTSLSMLACCR : 438
T.rubripes XP_003964129      : WTKNGVIRGQPSPLGAAVIDSPPQASSSASSSANDVSSMSTETTDPSSDPTVTSETDSSLDSHTSLGALACCR : 438
X.laevis BAB85483            : RAKNGVIRGQPAPLAQVQQ------------------------------------------------------ : 384
G.gullus XP_004942190        : RTKNGVIRGQPAPLAQVQQ------------------------------------------------------ : 384
M.musculus XP_006519093      : RTKNGVIRGQPSPLGAAMINGSQH--PSSSPSVNDMSSMSTDPT-------LASDTDSSLE--ASAGPLGCCR : 427
M.musculus XP_006519094      : RTKNGVIRGQPSPLGAAMINGSQH--PSSSPSVNDMSSMSTDPT-------LASDTDSSLE--ASAGPLGCCR : 427
H.sapiens NP_002741          : RTKNGVIRGQPSPLAQVQQ------------------------------------------------------ : 384
H.sapiens NP_620634          : RTKNGVIRGQPSPLAQVQQ------------------------------------------------------ : 384
```

FIGURE S2. Multiple sequence alignment of JNK1 in orange-spotted grouer with homologues in other vertebrates. Amino acid residues that are conserved in at least 80% sequences are shaded in dark, and similar amino acids are shaded in gray. The catalytic domains of Serine/Threonine kinase, c-Jun N-terminal kinase (STKc_JNK) is contained in the region marked with thin line above the amino acid sequences. While the domain of Serine/Threonine protein kinase (S_TKc) is marked with thick underline. The conserved “TPY” motif and Serine/Threonine protein kinases active site signature are boxed and pointed out. Other vertebrate JNK1s include *Danio rerio* (*D. rerio*), *Oryzias latipes* (*O. latipes*), *Takifugu rubripes* (*T. rubripes*), *Xenopus laevis* (*X. laevis*), *Gallus gallus* (*G. gallus*), *Mus musculus* (*M. musculus*) and *Homo sapiens* (*H. sapiens*). NCBI RefSeq or GenBank accession number of each species was listed on the right of the species name.
