# Supplementary material for: JNK1 Derived from Orange-Spotted Grouper, Epinephelus coioides, Involving in the Evasion and Infection of Singapore Grouper Iridovirus (SGIV)
Source: Front Microbiol. 2016 Feb 10;7:121. doi: 10.3389/fmicb.2016.00121 (PMC4748057; doi:10.3389/fmicb.2016.00121)
Supplement: Supplementary file 3 [file Image3.pdf]

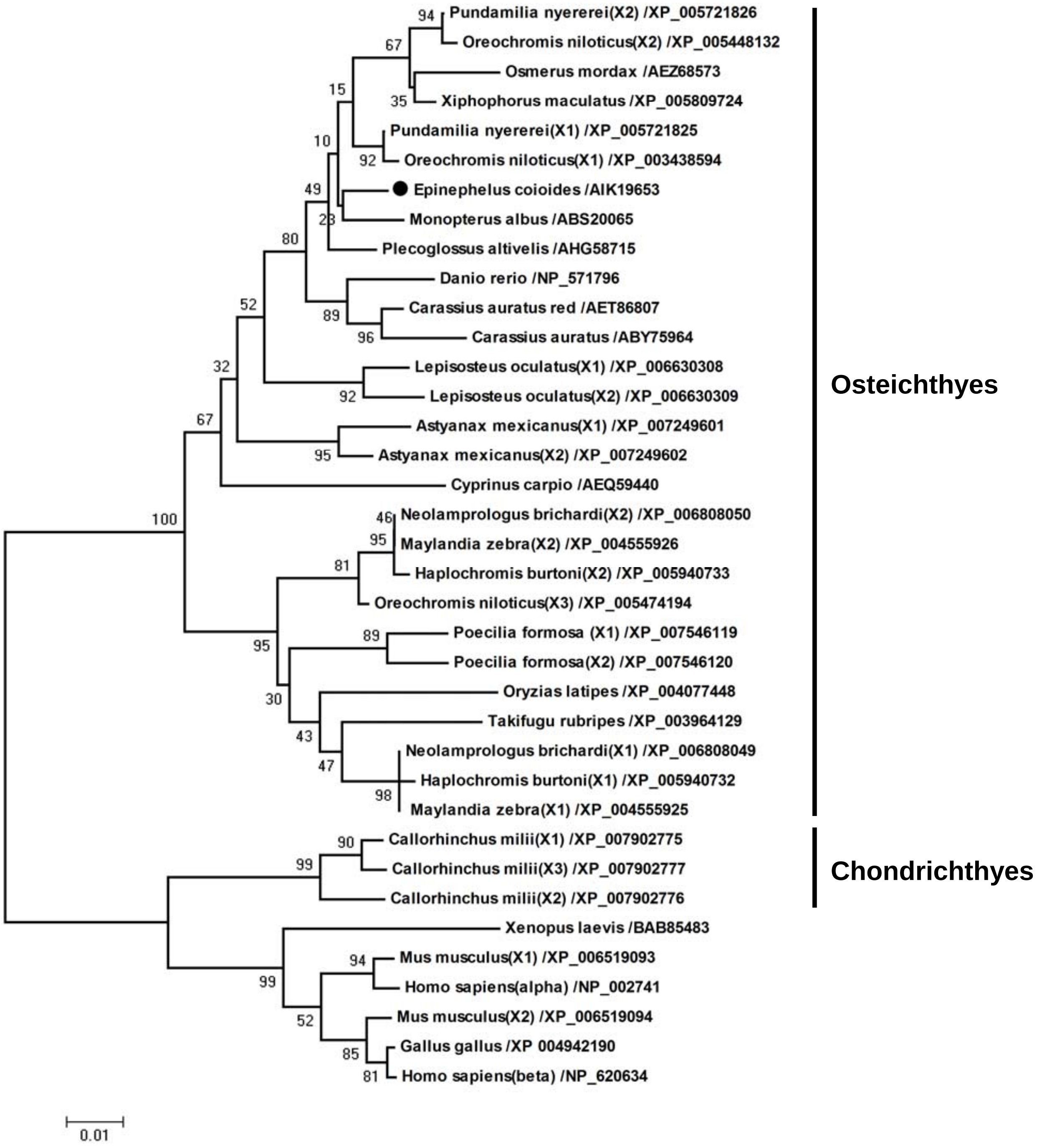

FIGURE S3. Phylogenetic analysis of Ec-JNK1 with other reported JNK1 in vertebrates. The phylogenetic tree was constructed according to the alignment of amino acid sequences by the neighbour-jointing method within MEGA 4.0. Numbers at branch nodes represent the bootstrap majority consensus values of 1000 replicates. NCBI RefSeq or GenBank accession number of each species was listed on the right of the species name.
